# A strong ‘filter’ effect of the East China Sea land bridge for East Asia’s temperate plant species: inferences from molecular phylogeography and ecological niche modelling of *Platycrater arguta* (Hydrangeaceae)

**DOI:** 10.1186/1471-2148-14-41

**Published:** 2014-03-04

**Authors:** Xin-Shuai Qi, Na Yuan, Hans Peter Comes, Shota Sakaguchi, Ying-Xiong Qiu

**Affiliations:** 1 Key Laboratory of Conservation Biology for Endangered Wildlife of the Ministry of Education, College of Life Sciences, Zhejiang University, Hangzhou, China; 2 Laboratory of Systematic & Evolutionary Botany and Biodiversity, College of Life Sciences, Zhejiang University, Hangzhou, China; 3 Department of Organismic Biology, Salzburg University, Salzburg A-5020, Austria; 4 Laboratory of Plant Evolution and Biodiversity, Department of General Systems Studies, Graduate School of Arts and Sciences, The University of Tokyo, Tokyo 153-8902, Japan

## Abstract

**Background:**

In East Asia, an increasing number of studies on temperate forest tree species find evidence for migration and gene exchange across the East China Sea (ECS) land bridge up until the last glacial maximum (LGM). However, it is less clear when and how lineages diverged in this region, whether in full isolation or in the face of post-divergence gene flow. Here, we investigate the effects of Quaternary changes in climate and sea level on the evolutionary and demographic history of *Platycrater arguta*, a rare temperate understorey shrub with disjunct distributions in East China (var. *sinensis*) and South Japan (var. *arguta*). Molecular data were obtained from 14 *P. arguta* populations to infer current patterns of molecular structure and diversity in relation to past (Last Interglacial and Last Glacial Maximum) and present distributions based on ecological niche modelling (ENM). A coalescent-based isolation-with-migration (IM) model was used to estimate lineage divergence times and population demographic parameters.

**Results:**

Combining information from nuclear/chloroplast sequence data with nuclear microsatellites, our IM analyses identify the two varieties as genetically distinct units that evolved in strict allopatry since the mid-Pleistocene, *c*. 0.89 (0.51–1.2) Ma. Together with Bayesian Skyeline Plots, our data further suggest that both lineages experienced post-divergence demographic growth, followed by refugial isolation, divergence, and in the case of var. *arguta* post-glacial admixture. However, past species distribution modelling indicates that the species’ overall distribution has not greatly changed over the last glacial cycles.

**Conclusions:**

Our findings highlight the important influence of ancient sea-level changes on the diversification of East Asia’s temperate flora. Implicitly, they challenge the notion of general temperate forest expansion across the ECS land bridge, demonstrating instead its ‘filter’ effect owing to an unsuitable environment for certain species and their biological (e.g., recruitment) properties.

## Background

Phytogeographers have long recognised the Sino-Japanese Floristic Region (SJFR) of East Asia as the world’s major centre of temperate plant diversity and endemism [1]. Much of the potential primary vegetation of this vast region is composed of warm-temperate deciduous (WTD) forest, as presently found scattered in mid-elevation subtropical (Central/South/East) China, predominant in low-elevation North China and the Korean Peninsula, and disjunctively distributed in the main islands of Japan [1,2]. Fossil pollen analyses have previously indicated that during the Last Glacial Maximum [LGM: *c.* 21,000–18,000 yr before present (BP)], the habitat of East Asian WTD forests in the northern parts of their range (e.g., in North China and North Japan) contracted, mainly in response to increased aridification [3,4]. However, palaeo-biome reconstructions suggest that these forests also expanded across the large expanses of continental shelf (*c.* 1 million km^2^) that emerged in the East China Sea (ECS) as a consequence of sea level lowering by *c.* 85–130/140 m during cold periods [5-7]. In consequence, it is now widely believed that a near continuous belt of WTD forests spanned the ECS continental shelf during the LGM (and possibly earlier cold periods), connecting presently disjunct populations in East China, South Japan, and the Korean Peninsula [2,8]. If correct, this land bridge may have served as a ‘dispersal corridor’ [9,10], allowing intermittent migration of most WTD forest-restricted plant species from the Asian mainland into Japan (or vice versa), and/or periodic secondary contact and gene flow among formerly isolated populations, possibly up until the last shelf submergence (*c.* 16,000–7,000 yr BP; [11]). However, there is accumulating but still scanty evidence that the ECS land bridge also acted as a ‘filter’ in selectively hampering or even preventing the dispersal of certain plant species, while allowing those able to tolerate the environmental conditions of this palaeo-landscape to disperse more freely (see below).

Support for the ‘recent dispersal corridor hypothesis’ comes from phylogeographic data of two wide-ranging and abundant WTD forest tree species of the SJFR (*Cercidiphyllum japonicum*[12] and *Kalopanax septemlobus*[13]). In both instances, there is a near lack of differentiation for chloroplast and/or nuclear DNA sequences across the ECS, consistent with ecological niche modelling (ENM) predicting large expanses of suitable habitat for each species on the ECS land bridge during the LGM. By contrast, a high level of genetic differentiation across the ECS has been identified in several rare understorey herbs and shrubs restricted to the mountainous WTD forests of East China and South Japan (*Croomia japonica*[14], *Platycrater arguta*/*Kirengeshoma palmata*[15,16] and *Ligularia hodgsonii*[17]). In all of these latter cases, the estimated times of trans-ECS divergence based on chloroplast (cp) DNA fall into the early-to-mid Pleistocene, suggesting that the ECS land bridge imposed a formidable barrier to dispersal during the last glacial cycle(s) (reviewed in [18]). However, all of these previous time estimates relied on a single locus, i.e., chloroplast (cp) DNA, which thus renders them subject to error from the inherent stochastic nature of the coalescent [19-21].

The aim of the present paper is to re-examine the evolutionary history of one of these understorey shrubs, *Platycrater arguta* Siebold & Zucc. (Hydrangeaceae), using a multi-locus approach. *Platycrater arguta*, the only species of this monotypic genus, is a small deciduous shrub endemic to the mountainous WTD forests of East China and South Japan, where respectively two varieties, var. *sinensis* and var. *arguta*, are recognized [22-24]. In the cpDNA study previously conducted [15], these taxa were found to comprise distinct phylogroups, whose likely vicariant origin was dated to the mid-Pleistocene (*c.* 0.89 Ma). Here we use a broader sampling of *P. arguta*, and present additional datasets of two nuclear DNA (nDNA) sequence markers (ITS, *Tpi*) and nuclear microsatellite (nSSR) loci to infer a more robust divergence and demographic history of this species. Specifically, our goals were (i) to provide a refined time-scale for the divergence of var. *sinensis* and var. *arguta* by fitting all four datasets (ITS, *Tpi*, nSSRs, cpDNA) to an ‘isolation with migration’ (IM) model [20,25]; (ii) to quantify the amount of post-divergence gene flow between them while accounting for potential changes in effective population sizes; and (iii) to model the species’ potential distributions in response to past climatic changes, specifically from the Last Interglacial (LIG/Eemian: *c.* 130,000–114,000 yr BP) [26] through the LGM to the present day. We attempted to reconcile these distribution patterns with our genetic data to determine the role of the exposed ECS shelf as a ‘corridor’ or ‘filter’ during lineage divergence at the time of the last glacial cycle(s). Finally, together with ENM, the added value of three nuclear data sets allowed for more confidence in the interpretation of the supposedly contrasting population histories of each variety than was derived previously from a single dataset (cpDNA) [15].

## Methods

### Plant material and sampling design

We sampled seven populations of each of var. *sinensis* and var. *arguta*, (272 individuals in total) covering the species’ entire range in East China and South Japan (Additional file 1: Table S1). Ten of the populations (100 individuals) were analysed previously for cpDNA [15], while four were newly sampled (C3–C5 and J2). As *P. arguta* is nested within a paraphyletic *Hydrangea* L., but with yet undefined sister species [27,28], we arbitrarily designated *H. chinensis* Maxim., collected from Yandang Mt. (China), as one of the outgroups together with other members of Hydrangeaceae (see below). Voucher specimens of this species and all sampled populations of *P. arguta* are stored at the Herbarium of Zhejiang University (HZU).

### Molecular protocols

Total genomic DNA was extracted from silica-dried leaf tissue by the cetyltrimethyl ammonium bromide (CTAB) method [29]. The entire internal transcribed spacer (ITS) region of nuclear ribosomal (nr) DNA was sequenced in 72 individuals of *P. arguta* (4 to 8 individuals per population; Additional file 1: Table S1) and one individual of *H. chinensis*, following the methods described in [14].

After preliminary screening of six low-copy number nuclear genes using primers developed by Strand et al. [30] (ADHX2F–4R, CAMX1F–2R, CHIX1F–4R, CHSX1F–2RN, GPDX7F–9R, TPIX4FN–6RN), we chose the triose-phosphate isomerase (*Tpi*) gene (TPIX4FN–6RN) for the full survey of 173 individuals (5–20 individuals per population, plus *H. chinensis*; Additional file 1: Table S1) because this gene region was single copy in most cases and proved to be sufficiently polymorphic [31]. Direct sequencing of *Tpi* was carried out using the same procedure as described in Zou *et al.*[32]. Chromatograms of ITS and *Tpi* with additive peaks were further analysed by inferring the identity of the two haplotypes within a heterozygote through haplotype subtraction [33,34]. When the chromatogram quality did not permit this procedure, PCR products were cloned using a pMD18-T vector system (Takara Biotechnology, Dalian, Liaoning, China) according to the manufacturer’s protocol. Six to ten clones were sequenced per individual when sequences contained two or more polymorphic sites. The two sequences of a heterozygote were separated by comparing sequences of the PCR product and the cloned sequences. All haplotype sequences have been deposited in GenBank [accession numbers for *P. arguta*: JQ978221–JQ978253 (ITS) and JQ978254–JQ978284 (*Tpi*); for *H. chinensis*: KF559183 (ITS) and KC853063 (*Tpi*)].

All 272 *P. arguta* individuals were genotyped at seven nuclear dinucleotide-repeat microsatellite loci (Pa1–Pa3, Pa5–Pa8) (Additional file 2: Table S2) according to the methods described in Qi *et al.*[35]. Fluorescently labelled PCR products (HEX or 6-FAM; Applied Biosystems, Foster City, CA, USA) were separated on a MegaBACE 1000 (GE Healthcare Biosciences, Pittsburgh, PA). Alleles were scored manually with the aid of GENETIC PROFILER 2.2 (GE Healthcare Biosciences) using the ET550-R size standard.

### Nuclear DNA sequence analyses

Sequences of ITS and *Tpi* were aligned and edited in GENEIOUS 4.8.2 [36]. For each gene region, we estimated haplotype diversity (*h*) and nucleotide diversity (*π*) for each population, for each variety, and for the whole data set. Tests for non-neutral evolution were conducted by computing Tajima’s *D*[37] and Fu & Li’s D* [38]. In addition, we estimated Fu’s *F*s [39] and Ramos-Onsins and Rozas’ *R*_2_[40] to detect population growth. Critical values for these statistics were obtained using 10^5^ coalescent simulations. Recombination was calculated as the minimum number of recombination events (*R*m) using the four-gamete test [41]. All of the above analyses were performed in DNASP 4.1 [42] and ARLEQUIN 3.11 [43].

Phylogenetic relationships of ITS and *Tpi* haplotype sequences were inferred using maximum parsimony (MP) and maximum likelihood (ML), with gaps (indels) treated as missing data. Heuristic MP searches were performed in PAUP* 4.0b10 [44] using the same settings as described in Qiu *et al*. [16]. The ML analysis was conducted under the GTR + Γ substitution model using RAXML 7.2.8 [45]. Node support was assessed using 1,000 ‘fast bootstrap’ replicates. The following species were chosen as outgroups: *Hydrangea chinensis* (ITS, *Tpi*) as well as *H. anomala* D. Don and *Schizophragma hydrangeoides* Siebold & Zucc. (only ITS; downloaded as GenBank accessions JF976651 and GU98303, respectively). The alignments and phylogenetic trees were deposited in TreeBASE (submission number 15354). In addition, we constructed haplotype networks for each dataset using the 95% statistical parsimony criterion implemented in TCS 1.21 [46]. For ITS, however, we had to increase the TCS connection limit to 50 steps to link the divergent networks of the two varieties. Indels were treated as single mutation events, and coded as substitutions (A or T). Population differentiation for unordered (*G*_ST_) and ordered (*N*_ST_) haplotypes were obtained with PERMUT to test whether *N*_ST_ is significantly larger than *G*_ST_ (1,000 random permutations), indicating the presence of phylogeographic structure [47]. Analyses of molecular variance (AMOVAs) were carried out in ARLEQUIN using *F-*statistics. Sequence variation was hierarchically partitioned between the two varieties, among populations within varieties, and within populations. The significance of all estimated fixation indices was tested using 10,000 permutations as described in Excoffier *et al.*[48].

### Nuclear microsatellite analyses

Measures of nSSR diversity were assessed for each population, and across all loci, by calculating in FSTAT 2.9.3 [49] the total number of alleles (*N*_A_), allelic richness (*R*_S_; standardized for 5 individuals using rarefaction), expected gene diversity (*H*_S_), and the inbreeding coefficient (*F*_IS_). Differentiation between populations was computed in ARLEQUIN using *R*_ST_[50], which assumes a stepwise mutation model (SMM; [51]), and thus may be more realistic for microsatellite data than other measures (e.g., *F*_ST_) based on the infinite alleles model (IAM).

Overall population structure was examined using the Bayesian clustering algorithm implemented in STRUCTURE 2.3 [52]. This program was run 10 times on individual multi-locus nSSR genotypes for a number of clusters (*K*), ranging from 1 to 14 (the number of localities), using a burn-in length of 50,000 and run length of 500,000 iterations. We used the admixture model without prior information on sample population membership and allowed allele frequencies to be correlated among clusters [53]. We plotted the posterior probability of the data [ln*P*(*D*)] and the ad hoc statistic Δ*K*[54] for determining the most likely *K*.

Hierarchical AMOVA was performed using *R*-statistics, and partitioned as described above. In order to detect genetic signatures of recent population bottlenecks in the nSSR dataset, we applied two tests implemented in BOTTLENECK 1.2.02 [55]: (i) Wilcoxon’s sign-rank test for heterozygote excess; and (ii) the ‘mode-shift test’ for detecting a shifted, rather than an equilibrium, L-shaped distribution of alleles.

### Divergence time, effective population sizes, and gene flow

We used the ‘isolation with migration’ (IM) coalescent model as implemented in IMA [25] to estimate population rate parameters (*Θ*) and effective population sizes (*N*_e_) of var. *sinensis* in China (*Θ*_C_), var. *arguta* in Japan (*Θ*_J_) and their common ancestral population (*Θ*_A_; *Θ* = 4*N*_e_u), as well as bidirectional migration rates (*m*_C–J_ and *m*_J–C_; *M* = *m*/u) and divergence times (*τ* = *t*u) between the two varieties. All parameters in the IM model are scaled by the neutral mutation rate (u). For this analysis we jointly employed the present nuclear datasets (ITS, *Tpi*, nSSRs) and previously obtained sequences of two cpDNA regions (*psb*A–*trn*H, *trn*D–*trn*E; [15]). Since IMA assumes no recombination within loci, the nDNA sequence data were analysed by using only maximally informative and nonrecombining blocks per individual as identified by the program IMGC [56].

Both nDNA and cpDNA sequence data were analysed under the HKY model rather than the infinite sites model (ISM) as we regularly found some sites with more than two changes in these data sets (note that HKY and ISM are the only models of nucleotide evolution implemented in IMA, whereby only HKY allows multiple changes at a site). Corresponding values of u were calculated as u = *μ*kg, where *μ* is the number of substitutions per site per year, k is the average sequence length under study in base pairs (excluding indels), and g is the generation time in years (i.e., age of first reproduction), which is five years in *P. arguta* as observed under cultivation [15]. As substitution rates for this species are unknown, we assumed the following mean values and confidence intervals (CI) in substitutions per site per year: ITS (for woody perennials), 2.15 × 10^−9^ (0.38–7.83 × 10^−9^) [57]; *Tpi*, 7 × 10^−9^ (0.5–3.0 × 10^−8^) [58,59]; and cpDNA, 1.52 × 10^−9^ (1.0–3.0 × 10^−9^) [58,60]. The nSSR loci were assumed to fit a stepwise model of mutation (SMM), with a mean mutation rate (μ) of 10^−5^ mutations per locus per year. Although the assumptions underlying this mutation rate are debatable (e.g., [61,62]), this rate falls close to the median of average values (*c.* 7.7 × 10^−4^) reported for a wider range of plant species [63-67], and has also been recently employed for other woody perennials [68,69]. The geometric average mutation rate of the four marker sets was used to rescale the IMA parameter estimates from the combined analysis. The inheritance scalars were set to 1 for the nuclear markers, and 0.5 for cpDNA, the latter value reflecting the expected effective population size (*N*_e_) of maternally inherited cpDNA in a hermaphroditic plant relative to an autosomal locus [70] (note that maternal inheritance of cpDNA has been demonstrated in Hydrangeaceae [71]). Markov chain Monte Carlo (MCMC) simulations were conducted for 10^7^ steps by using a linear heating scheme and 10 Metropolis-coupled chains with a burn-in period of 10^5^ steps. To verify convergence on the same parameter values, we ran this analysis three times with different random seeds. Only estimates whose posterior distribution dropped to zero within the prior intervals were trusted.

### Historical demography

To further examine the historical demography of each variety, we estimated the shape of the population growth function through time by constructing Extended Bayesian Skyline Plots (EBSPs) as implemented in BEAST 1.7.5 [72]. This coalescent-based, nonparametric Bayesian MCMC algorithm incorporates multi-locus data to reduce estimate errors associated with single genes and increases the power to detect demographic dynamics [73]. Analyses were performed for each variety across the sequence data sets (ITS, *Tpi*, cpDNA) assuming a strict molecular clock. The mutation rate priors for each locus were identical to those used for IMA. Based on the Akaike Information Criterion (AIC) as implemented in JMODELTEST [74], we selected the GTR + Γ and HKY substitution models for the nuclear and cpDNA sequences, respectively. Bayesian MCMC chains were run for 10 million generations, with a sampling frequency of every 1,000 generations, whereby the first 5,000 samples were discarded as burn-in. Convergence of the MCMC chains was inspected using TRACER 1.5 [75] by visually checking that effective sample size (ESS) for all relevant parameters were well above 200. Skyline plots were visualized using EXCEL.

### Present and past distribution modelling

To infer distributional changes of *P. arguta* since the last interglacial, we produced ENMs for three time periods (present, LGM, LIG) using the maximum entropy method implemented in MAXENT 3.2.1 [76]. In addition to the distribution records included in this study, records were sourced from the Chinese Virtual Herbarium (http://www.cvh.org.cn), the National Specimen Information Infrastructure of China (http://www.nsii.org.cn), the Kyoto University Herbarium (KYO), and Red Data Books for the Aichi and Mie Prefectures of Japan [77,78]. Based on a total of 59 records (China: 20; Japan: 39), a current distribution model was developed using six bioclimatic data layers (bio1, 12, 16, 17, 18, 19; WorldClim dataset, [79,13]) at 2.5 arc-min resolution. This model was then projected onto the set of climatic variables simulated by the Model for Interdisciplinary Research on Climate (MIROC) 3.2 [80] to infer the extent of suitable habitat during the LGM and the LIG [81-83,13]. The accuracy of each model prediction was then tested by calculating the area under the ‘Receiver Operating Characteristic (ROC) Curve’ (AUC; [84]). We note that the ENM for the LGM explicitly accounted for changes in palaeo-coastlines (−110 m than at present) and palaeo-climate surfaces on the exposed ECS continental shelf [7,85].

## Results

### Nuclear sequence characteristics

The ITS sequences of the 72 *P. arguta* individuals (14 populations) from East China (34) and South Japan (38) were aligned with a total length of 737 base pairs (bp), revealing 36 nucleotide substitutions and three 1-bp indels. Together, these 39 polymorphic sites yielded 33 ITS haplotypes (‘ribotypes’, H1–H33) (Additional file 3: Table S3). For the *Tpi* locus, only one or two distinct sequences were detected in each of the 173 individuals surveyed, 28 of which were found to be heterozygotes. In total, 31 haplotypes (T1–T31), ranging from 309 bp to 314 bp, were designated based on 39 substitutions and three small (≤ 5 bp) indels (Additional file 4: Table S4). None of the loci showed significant deviation from neutral expectations for Tajima’s *D* or Fu and Li’s D* at the species or variety levels (Additional file 5: Table S5). Demographic tests of Fu’s *F*s revealed negative but nonsignificant values, while *R*_2_ was consistently positive and significant, suggesting demographic growth (Additional file 5: Table S5). The minimum number of recombination events (*R*m) estimated for ITS and *Tpi* were 15 and 2, respectively.

### Genetic diversity, haplotype relationships and genetic structure at ITS and *Tpi*

**Figure 1:**
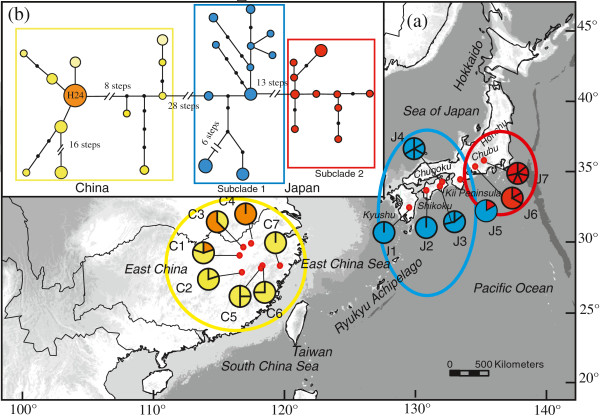
**Distribution of ITS ribotypes and the 95% plausible network of ribotypes in *Platycrater arguta*. (a)** Distribution of ITS ribotypes. All ribotypes, except for H24 denoted by orange colour, are population-specific, and represented with different colours corresponding to the major phylogroups. Population codes are identified in Additional file 1: Table S1. **(b)** TCS-derived network of genealogical relationships between the 33 ribotypes. The small, solid black circles represent missing ribotypes. The sizes of circles are approximately proportional to sample size (*n*), with the smallest circles representing *n* = 1 and the largest representing *n* = 10.

**Figure 2:**
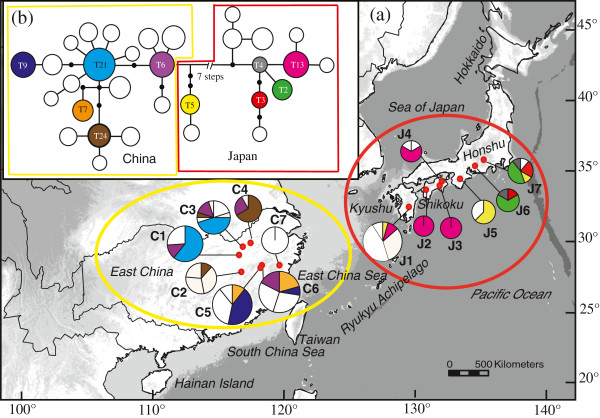
**Distribution of *Tpi *haplotypes and the 95% plausible network of *Tpi *haplotypes in *Platycrater arguta*. (a)** Distribution of *Tpi* haplotypes. Population codes are identified in Additional file 1: Table S1. The distributions of shared haplotypes are denoted by colour, while private haplotypes are white. **(b)** TCS-derived network of genealogical relationships between the 31 haplotypes. The small, solid black circles represent missing haplotypes. The sizes of circles are approximately proportional to sample size (*n*), with the smallest circles representing *n* = 1 and the largest representing *n* = 21.

There were high and comparable levels of total haplotype (*h*_T_) and nucleotide (*π*_T_) diversity at the species-level (ITS/*Tpi: h*_T_ = 0.96/0.92; *π*_T_ = 0.03923/0.02133), and the same was found for each variety (region), whereby each of the seven populations from East China (ITS/*Tpi: h*_T_ = 0.88/0.90; *π*_T_ = 0.0151/0.0108) and South Japan (ITS/*Tpi: h*_T_ = 0.95/0.87; *π*_T_ = 0.0188/0.0139) harboured broadly similar levels of diversity (Additional file 1: Table S1). Concordant with the previous cpDNA data [15], there was no sharing of haplotypes between China and Japan for either ITS (Figure 1a) or *Tpi* (Figure 2a).

**Figure 3:**
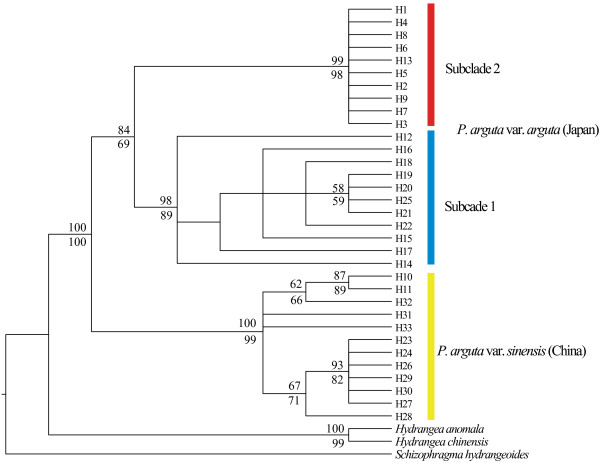
**Phylogenetic relationships of ITS ribotypes (H1–H33) of *Platycrater arguta*.** Individuals of *Hydrangea anomala, H. chinensis and Schizophragma hydrangeoides* were used as outgroup taxa. Phylogenetic analyses using maximum parsimony (MP) and maximum likelihood (ML) produced trees with the same topology regarding major lineages. Only the MP strict consensus tree is presented. Numbers above and below the branches indicate, respectively, MP and ML bootstrap values (> 50%).

For each of these nuclear markers, the tree topologies recovered from MP and ML analyses were similar, and only the MP strict consensus trees are shown. According to the ITS tree (Figure 3), rooted with three outgroup species, *P. arguta* was monophyletic (with MP/ML bootstrap values of 100/100%) and consisted of two major clades corresponding to var. *sinensis* from East China (100/99%) and var. *arguta* from South Japan (84/69%). In addition to these lineages previously identified by cpDNA, the ITS marker revealed two novel subclades, 1 (98/89%) vs. 2 (99/98%), predominant in the southern vs. northern parts of South Japan (Kyushu, Shikoku vs. central Honshu) but with apparent range overlap in the Kii Peninsula of south-central Honshu (population J5; Figure 1a). As can be seen from the unrooted ITS haplotype network (Figure 1b), the Chinese and Japanese haplotypes were separated from each other by 28 steps, and the two Japanese subclades by 13 steps.

**Figure 4:**
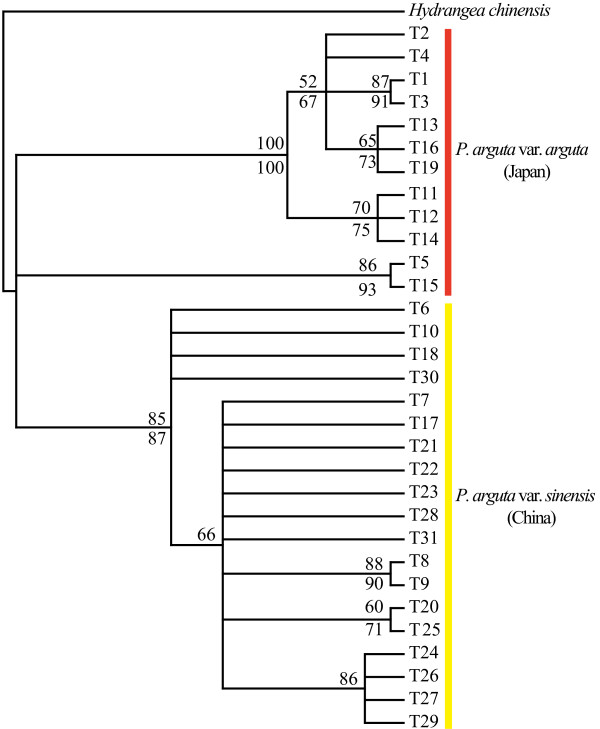
**Phylogenetic relationships of *Tpi *haplotypes (T1–T31) of *Platycrater arguta*. ***Hydrangea chinensis* was used as outgroup taxon. Phylogenetic analyses using maximum parsimony (MP) and maximum likelihood (ML) produced trees with the same topology regarding major lineages. Only the MP strict consensus tree is presented. Numbers above and below the branches indicate, respectively, MP and ML bootstrap values (> 50%).

The *Tpi* phylogeny, rooted with *Hydrangea chinensis* (Figure 4), had lower resolution than the ITS tree but still recovered all Chinese haplotypes as monophyletic (85/87%), and the same applied to the majority of those from Japan (100/100%). Both clades, however, formed a trichotomy with a deviant clade (86/93%) of the two remaining haplotypes (T5, T15) from Japan. These latter haplotypes, which showed no distinct geographic distribution (Figure 2a), occupied an intermediate position in the unrooted *Tp*i haplotype network (Figure 2b), yet with somewhat closer relationships to China rather than Japan (four versus nine mutational steps apart from each group).

For both ITS and *Tpi*, significant phylogeographic structure was obvious at the species-level and within each of the two varieties (*N*_ST_ > *G*_ST_, all *P* values < 0.01; Additional file 6: Table S6). Hierarchical AMOVA revealed that most of the total nuclear sequence variation was partitioned between them (ITS: 70.3%; *Tpi*: 66.7%; Table 1). Nevertheless, for each variety (*sinensis*/*arguta*), *N*_ST_ values were high at each nuclear sequence marker (ITS: 0.751/0.907; *Tpi*: 0.458/0.775) (Additional file 6: Table S6), reflecting that the majority of haplotypes were population specific (ITS: 32/33; *Tpi*: 26/31; see also Figures 1a and 2a).

**Table 1:** **Hierarchical analysis of molecular variance (AMOVA) of ITS and *Tpi *** **sequences and nSSRs from 14 populations of *Platycrater arguta *** **var. *sinensis *** **(East China) and var. *arguta *** **(South Japan)**

**Data sets/regional grouping of populations**	**Source of variation**	**d.f.**	**Sum of squares**	**Variance components**	**Variance explained (%)**	***F*- or *R*-statistics (*P*)**
ITS						
var. *sinensis* (China) vs. var. *arguta* (Japan)						
	Among groups	1	528.415	13.954	70.28	*F*_CT_ = 0.703**
	Among populations within groups	12	312.118	4.912	24.74	*F*_SC_ = 0.833**
	Within populations	58	57.315	0.988	4.98	*F*_ST_ = 0.950**
*Tpi*						
var. *sinensis* (China) vs. var. *arguta* (Japan)						
	Among groups	1	357.190	4.053	66.70	*F*_CT_ = 0.667**
	Among populations within groups	12	175.449	0.979	16.11	*F*_SC_ = 0.484**
	Within populations	187	195.305	1.044	17.19	*F*_ST_ = 0.828**
nSSRs						
var. *sinensis* (China) vs. var. *arguta* (Japan)						
	Among groups	1	25515.13	96.12	13.52	*R*_CT_ = 0.135**
	Among populations within groups	12	7106.99	178.93	25.17	*R*_SC_ = 0.290**
	Within populations	530	231038.92	435.92	61.31	*R*_ST_ = 0.390**

d.f., degrees of freedom; grouping of populations: Chinese group (populations C1–C7), Japanese group (populations J1–J7; for population codes see Additional file 1: Table S1, Supporting information); ** *P* < 0.001.

Estimators for *Tpi* and ITS were calculated based on the infinite alleles model (*F*-statistics) and those for nSSRs on the stepwise mutation model (*R*-statistics).

### Nuclear microsatellites

In 272 individuals from 14 populations of *P. arguta*, we detected a total of 220 alleles across 7 nSSR loci, with 17 to 48 alleles per locus. Average allele number (*N*_A_) was higher in China (mean: 66) than in Japan (mean: 43), but allelic richness (*R*_S_) and expected gene diversity (*H*_S_) were very similar (China: *R*_S_ = 4.69, *H*_S_ = 0.734; Japan: *R*_S_ = 4.68, *H*_S_ = 0.740; Additional file 1: Table S1). Within-population *F*_IS_ values ranged from −0.054 to 0.480, with an average of 0.246.

**Figure 5:**
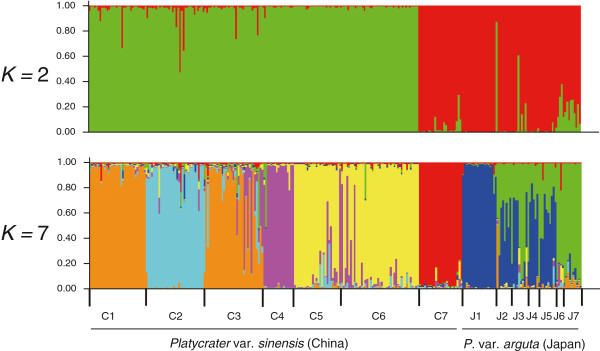
**The proportion of genetic clusters detected by STRUCTURE analysis for the model with peaks at *K*** **= 2 and *K*** **= 7.** The smallest vertical bar represents one individual. The assignment proportion of each individual into population clusters is shown along the y-axis. Note that STRUCTURE provided strongest support for *K* = 7, both when considering the probability of the data LnP(*D*) and Δ*K* (see text and Additional file 7: Figure S1).

The STRUCTURE analyses provided strongest support for *K* = 7, both when considering the probability of the data LnP(*D*) and Δ*K* (Additional file 7: Figure S1)*.* With *K* = 2, individuals from one population in China (C7) and Japanese populations formed a joint cluster, while the two varieties remained separate from *K* = 3 upwards (Figure 5). At *K* = 7, five clusters were recovered in China (var. *sinensis*) and two in Japan (var. *arguta*), where most populations (J2–J5) comprised individuals representing both clusters (Figure 5).

The overall *R*_ST_ was 0.340, reflecting strong genetic differentiation over all populations and within each variety (var. *sinensis*/*arguta*: 0.273/0.366). However, in contrast to ITS and *Tpi*, the total genetic variance partitioned between the two varieties was relatively low (13.5%; Table 1), suggesting pronounced allele sharing. Wilcoxon’s test and the complementary mode-shift test provided little evidence for recent population bottlenecks, except for three populations from Japan (J2, J4, J6) and partly depending on the test applied (Additional file 8: Table S7).

### Isolation-with-migration (IM) analyses

For this analysis, largest nonrecombining blocks of nDNA (ITS, *Tpi*) sequences were employed together with the nSSR markers and the previously obtained cpDNA sequences [15]. The maximum-likelihood estimates (MLEs) and the 90% highest probability density (HPD) intervals of the six IMA-derived parameters are shown in Table 2, and their marginal posterior probability (MPP) distributions are illustrated in Additional file 9: Figure S2. Based on the geometric average mutation rate calculated (5.79 × 10^−6^ mutations per locus per year), these parameter estimates were converted to absolute values of years or individuals (Table 2). The split between var. *sinensis* and var. *arguta* was dated to about 889,358 yr BP, with a 90% HPD interval ranging from 509,438 to 1,193,295 yr BP (Additional file 9: Figure S2a, Table 2). For the ancestral (*Θ*_A_) and descendant (*Θ*_C_ and *Θ*_J_) population rate parameters, both var. *sinensis* (*N*_C_ = 1.13 × 10^5^) and var. *arguta* (*N*_J_ = 6.00 × 10^4^) experienced a marked increase in effective population size (*N*_e_) relative to that of their common ancestor (*N*_A_ = 2.73 × 10^4^) (Additional file 9: Figure S2b, Table 2). Peak posterior estimates of post-divergence migration were effectively zero in both directions (*m*_C–J_ = *m*_J–C_ = 0.005; Additional file 9: Figure S2c, Table 2).

**Table 2:** **Maximum-likelihood estimates (MLEs) and lower and upper bounds of the 90**% **highest posterior density intervals (HPD90**_**Lo **_**and HPD90**_**Hi**_**, respectively) of demographic parameters of *Platycrater arguta *from the IMA analysis of multi-locus data (cpDNA, ITS, *Tpi*, nSSRs)**

**Estimates**	***Θ***_**C**_	***Θ***_**J**_	***Θ***_**A**_	***m***_**C-J**_	***m***_**J-C**_	***t***	***N***_**C**_	***N***_**J**_	***N***_**A**_	**2N**_**C**_**M**_**C-J**_	**2N**_**J**_**M**_**J-C**_	***T *(years** **BP)**
MLE	106.596	56.652	25.730	0.005	0.005	5.150	113047	60080	27287	0.00005	0.00003	889,358
HPD90_Lo_	55.136	29.992	11.027	0.005	0.005	2.950	58472	31807	11694	0.00003	0.00002	509,438
HPD90_Hi_	231.570	109.971	69.839	0.085	0.085	6.910	245584	116626	74065	0.00010	0.00005	1193,295

Population rate parameters *Θ*_C_, *Θ*_J_, and *Θ*_A_ refer to the scaled effective population sizes (*N*_e_) of var. *sinensis* (East China), var. *arguta* (South Japan), and the ancestral population, respectively. *m*_C-J_ and *m*_J-C_ are the scaled migration rates forward in time from var. *sinensis* to var. *arguta* and vice versa. M_C-J_ and M_J-C_ are the probabilities of migration from var. *sinensis* to var. *arguta*, per gene copy per generation and vice versa. 2N_C_M_C-J_ and 2N_J_M_J-C_ are the effective migration rates (number of migrants per generation). *t* is the time since ancestral population splitting in mutational units.

All estimates include the per gene mutation rate u, which is equal to the geometric mean of the mutation rates of all the loci. *Θ*_C_, *Θ*_J_, *Θ*_A_, *m*_C-J_, *m*_J-C_, and *t* are scaled by the mutation rate, while *N*_C_, *N*_J_, *N*_A_, 2N_C_M_C-J_, 2N_J_M_J-C_ and *T* are scaled by individuals or years.

### Bayesian skyline plot analysis of historical demography

The EBSPs show that both varieties maintained low but stable effective population sizes (*N*_e_) up to approximately 0.4–0.5 Ma, and appear to have experienced a constant increase since (Additional file 10: Figure S3). While depicting a demographic trend over time, this analysis, however, cannot precisely estimate *N*_e_ because of the very broad confidence intervals (see Additional file 10: Figure S3).

### Present and past ecological niche models

**Figure 6:**
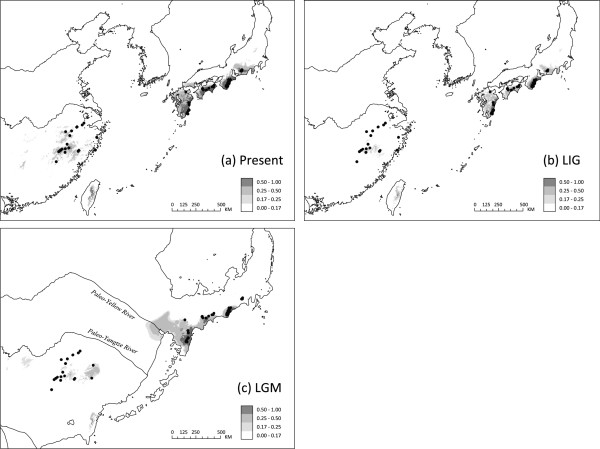
**Modelled climatically suitable areas of *Platycrater arguta *in East Asia at different times. (a)** the present; **(b)** the Last Interglacial (LIG/Eemian: *c.* 130,000–114,000 yr BP); and **(c)** the Last Glacial Maximum (LGM: *c.* 21,000–18,000 yr BP). The current ecological niche model was established with six bioclimatic data layers on the basis of 59 sites of presence records of the species (black dots) using MAXENT 3.2.1 [76] and then projected onto a set of climatic variables simulated by MIROC 3.2 [80] to infer the extent of suitable habitats during the LGM and the LIG (see text). The map in **(c)** reflects changes in coastline and shelf exposition during the LGM due to lowered sea level (−110 m than at present; e.g., [7]). Yellow River and Yangtze Rive plaeo-channels in the exposed East China Sea basin are modified after [86]. The logistic value of habitat suitability is shown according to the grey-scale bars.

The AUC value for the current potential distribution of *P. arguta* was 0.981, indicating a good predictive model performance. For the present (Figure 6a), these predicted areas mainly include the species’ known distribution ranges in East China (Wuyi/Yandang Mts.) and South Japan (Kyushu, Shikoku, Kii Peninsula/south-central Honshu, and the Pacific Ocean side of central Honshu). Further suitable habitat is predicted in central-eastern Taiwan, but where the species is not known to occur. During the LIG (Figure 6b), the species’ potential range in China was apparently reduced compared to the present. Also in the LGM (Figure 6c) only small and disjunct areas are predicted as suitable in the Wuyi/Yandang Mts. In contrast, in Japan, the species’ current range is more or less similar to that during the LIG (Figure 6b), while during the LGM suitable habitat apparently contracted to the south (Kyushu, Shikoku) and the more northerly located Kii Peninsula (Figure 6c). Most strikingly, our LGM distribution model indicates almost no hospitable areas on the exposed ECS continental shelf, excepting those in the far east (i.e., extending from Kyushu to the delta region of the palaeo-Yellow River).

## Discussion

### Intraspecific divergence and post-divergence gene flow

Our results provide strong evidence that the two recognized varieties of the WTD understorey shrub *P. arguta* in East China (var. *sinensis*) and South Japan (var. *arguta*) comprise reciprocally exclusive haplotypes for both ITS and *Tpi* (Figures 1 and 2). This is concordant with patterns for cpDNA [15] and consistent with the hypothesis that Chinese and Japanese conspecifics diverged over multiple glacial periods without inter-population gene flow. However, relationships inferred from the STRUCTURE analysis of nSSRs with *K* = 2 seem to conflict with this interpretation, as one Chinese population (C7) clusters with those from Japan (Figure 5). Given the congruence between nuclear and chloroplast sequence data, the sharing of nSSR alleles most likely reflects homoplasy and/or incomplete lineage sorting rather than admixture through rare long-distance dispersal and/or migration across the ECS land bridge. In fact, our IMA analysis of combined multi-locus data (cpDNA, ITS, *Tpi*, nSSRs) recovered only close to zero signals of bidirectional post-divergence gene flow between the two varieties following their estimated divergence in the mid-Pleistocene, *c.* 0.89 Ma (90% HPD: 1.2–0.51 Ma). Although this timing perfectly matches the respective point estimate drawn from cpDNA alone [15], single-locus cpDNA analysis suffered from a lack of convergence of the lineage divergence parameter (*t*), a problem that has been overcome with the present multi-locus approach (Additional file 9: Figure S2).

### Broad-scale biogeographic history of *P. arguta*

According to Kimura [86,87], there were three main stages of land connections between the Eurasian mainland and the Japanese/Ryukyu Islands since the Late Miocene, with most of the ECS sea-floor exposed at about 7.0–5.0 Ma, 2.0–1.3 Ma, and 0.2–0.015 Ma, whereby the latter interval includes land bridge formations in both the penultimate (Riss) and last (Würm) glacial periods [88-90]. Our estimated divergence time and its HPD intervals fall into the period (1.3) 1.0–0.2 Ma, the so-called ‘Ryukyu Coral Sea Stage’, when sea level rose tremendously and most formerly exposed land area submerged [87]. Based on these palaeo-data, and with no genetic indication of long-distance dispersal, the disjunct distribution of *P. arguta* across the ECS is implied to have resulted via vicariance, that is, the species was probably more widely distributed on the ECS land bridge in the early Pleistocene, around 2.0–1.3 Ma, while divergence was subsequently driven by population contraction and extinction through land-bridge submergence.

Unfortunately, it is not possible to test this ‘early-Pleistocene expansion’ hypothesis further using ENM, as no proxy-climate records are currently available for time periods earlier than the Last Interglacial (LIG) [91]. However, loess deposit and marine δ^18^O records indicate that both aridification and the influence of cold and dry winter monsoons in the northern subtropical areas of East Asia were less extensive before the mid-Pleistocene climate transition (MTP), *c.* 0.85 Ma [92]. Afterwards, global ice volume drastically increased along with a change in the dominant orbital cycle from 41,000 to 100,000 years, resulting in drier and colder climates during glacial periods [93]. Given that *P. arguta* is a moisture-dependent and drought-sensitive shrub, early-Pleistocene environmental conditions may have still favoured a more continuous distribution on the ECS land bridge, whereas subsequent glacials would have prevented the migration of individuals between China and Japan. Indeed, our LGM distribution model for *P. arguta* essentially shows no suitable habitat areas on the exposed ECS basin, with the exception of the delta region of the palaeo-Yellow River (Figure 6c). We therefore conclude that the ECS land bridge acted as a formidable barrier to the dispersal of *P. arguta* during the LGM and earlier cold periods of the Late Pleistocene. However, additional factors, other than climate-related niche requirements, may have had a role in preventing, or at least hampering such dispersal. This is because similar (i.e., genetic and ENM) evidence suggests that the ECS land bridge served as an intermittent route of range expansion for most of the Late Pleistocene to counteract isolation between Chinese and Japanese populations of two widespread WTD forest tree species, namely *Cercidiphyllum japonicum*[12] and *Kalopanax septemlobus*[13]. Both are tall canopy trees with long generation times, large effective population sizes and high seed production [94,95], and additional traits generally considered important for population recovery, such as a generalist (i.e., wind) pollination/dispersal system (*C. japonicum*) and vegetative reproduction (*C. japonicum, K. septemlobus*). Similar traits are largely absent or only moderately developed in *P. arguta* (except for its wind-dispersed seed); the same applies to other rare and narrowly distributed understorey plants of the same forest biome with likewise ancient (early-to-mid Pleistocene) genetic breaks across the ECS (*Croomia japonica*: [14]; *Kirengeshoma palmata*: [16]; *Ligularia hodgsonii*: [17]). Moreover, in forest habitats particularly, one should not dismiss the effects related to edges (e.g., abundance to animal pollinators and/or dispersers), which may either increase or decrease seed production and recruitment [96-98]. Hence, species in the forest interior, such as *P. arguta* and other understorey plants, should be negatively affected by fragmentation; by contrast, species using edge or transitional habitats, such as the riparian-dwelling *C. japonicum* and the semi-invasive *K. semptemlobus*, may be favoured by fragmentation [96,97]. We therefore propose that (i) range expansion in response to the formation of the glacially exposed ECS land bridge is species-specific; and (ii) predictions on the effects of this ephemeral land bridge, as ‘corridor’ or ‘filter’, have to account not only for habitat preferences *per se* but also for other biological features of each species, especially its recruitment properties.

### Inferences of historical demography and range dynamics in *P. arguta*

Our results provide strong evidence that both var. *sinensis* and var. *arguta* underwent past population growth following their inferred sundering in the mid-Pleistocene. This is supported by significantly positive *R*_2_ statistics for both ITS and *Tpi* (Additional file 5: Table S5), and is also evident from the joint analysis of the four genetic data sets (cpDNA, ITS, *Tpi,* nSSRs) with IMA, suggesting a somewhat larger effective population size (*N*_e_) in each variety compared to their ancestral population (Additional file 9: Figure S2b, Table 2). We caution, however, that this estimate of ancestral *N*_e_ probably reflects post-vicariant conditions, and thus is biased downward, as complex population dynamics within the ancestral population, such as contractions and extinctions, are not accounted for by IMA [99]. Nonetheless, analyses of the combined nuclear and plastid sequence data with EBSPs were consistent in showing a strong increase of *N*_e_ in each variety from about 0.4–0.5 Ma onwards (Additional file 10: Figure S3). This is very similar to the point estimates of expansion times inferred from mismatch analyses of cpDNA alone, that is, *c.* 0.43 and 0.45 Ma for var. *sinensis* and var. *arguta*, respectively [15]. Hence, the present results support our previous notion that this near-synchronized population growth may have been triggered by climate change at the beginning of China’s ‘Penultimate Interglacial Period’ (*c.* 0.46–0.33 Ma; [100]). This also accords with palaeo-climate data, suggesting that during interglacials since the mid-late Pleistocene (*c.* 1.0–0.78 Ma) the warm, wet East Asian summer monsoons have intensified [101-103]. In consequence, this may have also led to an increase of suitable habitats for *P. arguta* throughout the warmer periods of the Late Quaternary. Partly consistent with this, the ENM shows larger and more contiguous distributions at least in northern South Japan [i.e. Kii Peninsula and (south-)central Honshu] at both the LIG and the present compared to the LGM (Figure 6). Although suitable habitats may have shrunk in East China at the LIG (Figure 6b), and in both regions during the LGM (Figure 6c), we found no direct (EBSP) evidence for population decline (Additional file 10: Figure S3) and recent population bottlenecks (Additional file 1: Tables S1; Additional file 5: Tables S5). Nonetheless, the ENM suggests that potential glacial refugia in East China were more strongly affected by small-scale fragmentation compared to the situation in South Japan (Figure 6c) in general, and its southern parts (Shikoku, Kyushu) in particular, where such refugia might have even existed in nearby shelf areas. This inter-regional difference in glacial habitat suitability most likely reflects differences in topography and (micro-)climate, and should have differing consequences for population genetic structure and evolutionary history (see below).

### Contrasting Late Quaternary evolutionary histories of *P. arguta* in China and Japan

In var. *sinensis*, there is a marked subdivision between populations in nuclear genes (ITS, *Tpi*, nSSRs) (Table 1), along with high haplotypic and allelic diversity (Additional file 1: Table S1), and the same holds true for cpDNA [15]. Together with our STRUCTURE result of five principal nSSR gene pools in this variety (Figure 5), all of these data indicate long-term population persistence and isolation over multiple glacial/interglacial cycles. With no indication of latitudinal range shifts, past population growth in var. *sinensis* (see above) may also reflect, at least in part, repeated downhill shifts in elevation range during glacials, perhaps by tracking favourable humidity conditions as imposed by the East Asian monsoon in areas of high relief [104]. Over climatic cycling, such contiguous but localized range shifts along elevation gradients would have minimized bottlenecking and loss of genetic diversity, ultimately resulting in strong population subdivision [105].

In Japan, patterns of cpDNA diversity in var. *arguta* were previously interpreted to indicate southward retreat during glacials (to Kyushu and Shikoku) followed by expansion northward (up to central Honshu) during inter-/post-glacials [15]. However, the current nuclear diversity and ENM data cast doubt on the validity of the ‘expansion-contraction’ (EC) model for var. *arguta*. For example, nuclear genetic diversity within var. *arguta* populations is more or less evenly spread throughout the distribution (Additional file 1: Table S1), and neither haplotypic (*h*_S_) diversity (ITS, *Tpi*) or allelic richness (*R*_s_; nSSRs) show a significantly negative relationship with latitude (*P* = 0.33–0.58; data not shown), as would be expected under a scenario of south-to-north (re-)colonization [106,107]. In addition, the ENM for the LGM indicates suitable habitats not only in the south (Kyushu, Shikoku, and nearby shelf areas) but also, more disjunctively, in the Kii Peninsula of south-central Honshu (Figure 6c). By contrast, large areas of contiguous suitable habitats were likely present at the LIG (Figure 6b), suggesting that opportunities for admixture occurred repeatedly during inter-/postglacial times, unless hampered by the spread of evergreen forest [1,4].

Both of these latter predictions are basically bone out by the current nuclear data, even though each marker suggests a somewhat different historical scenario. Thus, the identification of two separate ITS lineages in Kyushu/Shikoku vs. central Honshu, with an apparent overlap of range in the Kii Peninsula (pop. J5; Figures 1a and 3), is strongly suggestive of a two-refugia scenario and a narrow contact zone between the two lineages. Interestingly, this putative contact region coincides with a well-known inter- and intraspecific suture zone of Japan’s temperate flora and fauna [14,108,109,12]. On the other hand, the *Tpi* data are more consistent with a multiple-refugia scenario, that is, haplotypes are largely restricted to particular (sub)regions and/or populations, while still showing a relatively small amount of shared polymorphisms, mainly (but not exclusively) among adjacent sites (Figure 2a). Finally, the nSSRs reveal extensive admixture in most populations (J2–5), excepting those in Kyushu (J1) and central Honshu (J6, J7; Figure 5), suggesting that, in marked contrast to ITS, the contact area is much wider towards the south.

This discordance among patterns of genetic structure observed in the four genetic data sets makes it difficult to specify exactly which historical processes occurred in var. *arguta*. This discordance (even among nuclear genes and relative to cpDNA) is not readily explicable by a single difference in marker properties [e.g., mutation rates, transmission genetics, effective population sizes, concerted evolution (only ITS)], but likely results from a combination of marker features, with both incomplete lineage sorting and secondary admixture acting at different temporal and spatial scales [83]. Taken together, we suggest that the genetic and ENM patterns found in var. *arguta* reflect a relatively ancient north–south vicariant event during glacials (sundering populations in central Honshu), followed by more recent climate-induced cycles of range contraction and expansion/admixture, with the latter producing more shallow patterns of divergence in more southerly areas (Kyushu, Shikoku, Kii).

## Conclusions

This is the first study providing firm evidence that the ECS land bridge acted as a ‘filter’ during the last glacials in selectively preventing the dispersal of certain plant species of WTD forest, such as *P. arguta*, even though a near continuous belt of this forest biome is thought to have covered this land bridge during the LGM [2]. Our data emphasize the species-specific recruitment and range expansion response of WTD forest-dwellers to the formation of the ECS land bridge, and caution against an uncritical interpretation of reconstructed palaeo-forest biome data as guides of past range dynamics of constituent species populations [8].

In addition, this multi-locus study strongly supports the two varieties of *P. arguta* in East China (var. *sinensis*) and South Japan (var. *arguta*) as genetically distinct units that evolved in strict allopatry since the mid-Pleistocene, which concurs with previous findings inferred from cpDNA alone [15]. However, the combination of genetic structures from both nuclear and cytoplasmic loci can better depict the history of populations, demonstrating that (i) each lineage has undergone refugial isolation and divergence; and (ii) var. *arguta* likely experienced post-glacial admixture across a well-known suture zone. Yet, the extent of the species’ overall distribution does not seem to have greatly changed over the last glacial-interglacial cycles.

### Availability of supporting data

The sequence alignments and phylogenetic trees were deposited in TreeBASE (http://treebase.org/treebase-web/home.html) under the submission number 15354. Sequence data used in this study have been deposited in GenBank (JQ978221––JQ978284, KF559183, KC853063).

## Competing interests

The authors declare that they have no competing interests.

## Authors’ contributions

YXQ conceived the ideas; SS and XSQ contributed to the sampling; XSQ and YN collected and analysed the molecular data and drafted the manuscript. SS performed the ENM. The manuscript was written by HPC and YXQ. All authors read and approved the final manuscript.

## Supplementary Material

Additional file 1: Table S1 Geographic and genetic characteristics of 14 populations of *Platycrater arguta* from East China (var. *sinensis*: C1–C7) and South Japan (var. *arguta*; J1–J7) surveyed for nDNA (*Tpi,* ITS) sequence and nSSR variation. Click here for file

Additional file 2: Table S2 Characteristics of 7 microsatellite loci developed for *Platycrater arguta*. Shown for each locus are the locus name, the forward (F) and reverse (R) primer sequence, repeat motifs, the optimized annealing temperature (*T*a), allele size ranges, the GenBank accession number. Click here for file

Additional file 3: Table S3 ITS sequence polymorphism detected in *Platycrater* at ITS1 and ITS2 (the 5.8S excluded) regions and identifying 33 haplotypes (H1−H33). A dash (−) denotes a single nucleotide indel. Note that poly-A or poly-T stretches were excluded from analysis. Click here for file

Additional file 4: Table S4 The sequence polymorphism detected in *Platycrater* at *Tpi* gene region. The 31 haplotypes were denoting as T1−T 31. A dash (−) denotes a single nucleotide indel. Click here for file

Additional file 5: Table S5 Tests of neutrality and population growth for nDNA (ITS, *Tpi*) sequence variation in *Platycrater arguta* and each variety. Click here for file

Additional file 6: Table S6 Mean estimates (± SE) of gene diversity within populations (*h*_S_), total gene diversity (*h*_T_), and population differentiation for unordered (*G*_ST_) and ordered (*N*_ST_) haplotypes of ITS and *Tpi* in *Platycrater arguta* and each variety. 1,000 random permutations were performed to test whether *N*_ST_ is significantly larger than *G*_ST_. Click here for file

Additional file 7: Figure S1 (a) The posterior probability of the nSSR data on 14 *Platycrater arguta* populations given *K* clusters, obtained through 10 runs of the STRUCTURE algorithm (Falush *et al*. [53]). (b) The corresponding Δ*K* statistic (Evanno *et al*. [54]) showing peaks at *K* = 2 and *K* = 7, indicating that those are the best solutions for *K* given the data. Click here for file

Additional file 8: Table S7 BOTTLENECK analyses of nSSRs for 14 populations of *Platycrater arguta* from East China (var. *sinensis*: C1–C7) and South Japan (var. *arguta*; J1–J7). *P* values are shown for Wilcoxon’s signed rank test, under both the stepwise mutation model (SMM) and the two-phase mutation model (TPM), along with the distribution shape of alleles inferred from the mode-shift test. Population codes are identified in Table S1. Click here for file

Additional file 9: Figure S2 Marginal posterior probability (MPP) distributions of six IMA-derived model parameters for *Platycrater arguta* var. *sinensis* (East China) and var. *arguta* (South Japan). (a) The time (*t*) since ancestral population splitting in mutational units. (b) The scaled effective population sizes (*Θ*) of var. *sinensis* (*Θ*_C_), var. *arguta* (*Θ*_J_), and the ancestral population (*Θ*_A_). (c) The scaled migration rates forward in time from East China to South Japan (*m*_C-J_), and vice versa (*m*_J-C_). Click here for file

Additional file 10: Figure S3 Extended Bayesian Skyline Plots (EBSPs), inferred from cpDNA and nDNA (ITS, *Tpi*) sequence variation and depicting changes in effective population size (*N*_e_) as a function of time for (a) *Platycrater arguta* var. *sinensis* (East China) and (b) var. *arguta* (South Japan). The thick solid black line is the median estimate, and the area delimited by the upper and lower grey lines represents the HPD 95% confidence intervals for *N*_e_. Click here for file
