# Supplementary material for: A strong ‘filter’ effect of the East China Sea land bridge for East Asia’s temperate plant species: inferences from molecular phylogeography and ecological niche modelling of Platycrater arguta (Hydrangeaceae)
Source: BMC Evol Biol. 2014 Mar 4;14:41. doi: 10.1186/1471-2148-14-41 (PMC4015774; doi:10.1186/1471-2148-14-41)
Supplement: Additional file 1: Table S1 — Geographic and genetic characteristics of 14 populations of Platycrater arguta from East China (var. sinensis: C1–C7) and South Japan (var. arguta; J1–J7) surveyed for nDNA (Tpi, ITS) sequence and nSSR variation. [file 1471-2148-14-41-S1.docx]

**Additional file 1: Table S1.** Geographic and genetic characteristics of 14 populations of *Platycrater arguta* from East China (var. *sinensis*: C1–C7) and South Japan (var. *arguta*; J1–J7) surveyed for nDNA (*Tpi,* ITS) sequence and nSSR variation.

| Region/  population code | Locality | Lat (°N), Long (°E) | Alt  (m) | *n* (*Tpi*/ITS/nSSR) | *Tpi* |  |  | ITS |  |  | nSSRs | | | | |  |
| --- | --- | --- | --- | --- | --- | --- | --- | --- | --- | --- | --- | --- | --- | --- | --- | --- |
|  |  |  |  |  | *h* | π |  | *h* | π |  | *N*_A_ | *R*_S_ | *H*_S_ | | *F*_IS_ | |
| East China | | | | | | | | | | | | | | | |  |
| C1 | Mt. Matoushan, Jiangxi Province | 27°48', 117°30' | 600–900 | 28/5/31 | 0.569 | 0.0028 |  | 0.700 | 0.0020 |  | 69 | 4.78 | 0.739 | -0.054 | |  |
| C2 | Mt. Wuyishan, Fujian Province | 27°27', 118°01' | 600–900 | 17/5/33 | 0.765 | 0.0078 |  | 0.400 | 0.0020 |  | 75 | 4.96 | 0.756 | 0.193 | |  |
| C3 | Shuangzhen Country, Jiangxi Province | 27°56', 117°22' | 260 | 20/8/32 | 0.695 | 0.0043 |  | 0.536 | 0.0009 |  | 85 | 5.5 | 0.800 | 0.152 | |  |
| C4 | Mt.Wufu, Jiangxi Province | 28°06', 118°07' | 480 | 12/4/17 | 0.409 | 0.0065 |  | 0.000 | 0 |  | 50 | 4.75 | 0.781 | 0.269 * | |  |
| C5 | Mt. Fengyangshan, Zhejiang Province | 27°51', 119°09' | 700 | 28/4/24 | 0.690 | 0.0112 |  | 0.833 | 0.0061 |  | 49 | 3.81 | 0.638 | 0.319 * | |  |
| C6 | Mt. Baishanzu, Zhejiang Province | 27°03', 119°09' | 700–1,400 | 29/4/45 | 0.798 | 0.0112 |  | 0.500 | 0.0008 |  | 67 | 4.31 | 0.728 | 0.172 | |  |
| C7 | Mt. Yandangshan, Zhejiang Province | 28°02', 121°00' | 400–600 | 12/4/24 | 0 | 0 |  | 0.000 | 0 |  | 65 | 4.73 | 0.694 | -0.003 | |  |
| Regional mean | – | – | – | – | 0.561 | 0.0063 |  | 0.424 | 0.0018 |  | 66 | 4.69 | 0.734 | – | |  |
| Regional total | – | – | – | 141/34/206 | 0.903 | 0.0108 |  | 0.881 | 0.0151 |  | 192 | 27.42 | 0.856 | – | |  |
| South Japan | | | | | | | | | | | | | | | |  |
| J1 | Mt. Tsubaki-yama and Mt. Kaichigo, Miyazaki Prefecture, Kyushu | 31°26', 131°16' | 250 | 22/4/19 | 0.706 | 0.0062 |  | 0 | 0 |  | 53 | 4.09 | 0.590 | 0.299* | |  |
| J2 | Ehime Pref., Shikoku, Japan | 32°43', 131°11' | 550 | 4/4/5 | 0 | 0 |  | 0.667 | 0.0033 |  | 32 | 4.57 | 0.796 | 0.211* | |  |
| J3 | Nishiguma Valley, Kami City, Kochi Pref., Shikoku | 33°33', 133°31' | 690 | 5/5/10 | 0 | 0 |  | 0.8 | 0.0037 |  | 49 | 4.98 | 0.799 | 0.374* | |  |
| J4 | Nakatsu Valley, Niyodogawa Town, Kochi Pref., Shikoku | 33°35', 133°10' | 170 | 6/6/8 | 0.600 | 0.0021 |  | 0.933 | 0.0053 |  | 38 | 4.51 | 0.790 | 0.480* | |  |
| J5 | Kuwannokidani Valley, Shingu City, Mie Pref., Honshu/ Kii Peninsula | 33°43', 135°59' | 130 | 8/6/10 | 0.536 | 0.0017 |  | 0.333 | 0.0111 |  | 45 | 4.59 | 0.729 | 0.196 | |  |
| J6 | Shirakurakyo, Hamamatsu City, Shizuoka Pref., Honshu/Chubu | 34°59', 137°47' | 200 | 6/6/5 | 0.600 | 0.0038 |  | 0.600 | 0.0019 |  | 38 | 5.43 | 0.796 | 0.283* | |  |
| J7 | Sakuma, Hamamatsu City, Shizuoka Pref., Honshu/Chubu | 35°06', 137°49' | 200 | 9/7/9 | 0.806 | 0.0121 |  | 0.952 | 0.0047 |  | 43 | 4.56 | 0.683 | 0.163 | |  |
| Regional mean | – | – | – | – | 0.464 | 0.0038 |  | 0.612 | 0.0043 |  | 43 | 4.68 | 0.740 | – | |  |
| Regional total | – | – | – | 60/38/66 | 0.871 | 0.0139 |  | 0.954 | 0.0188 |  | 128 | 18.28 | 0.825 | – | |  |
| Species mean | – | – | – | – | 0.503 | 0.0056 |  | 0.544 | 0.0044 |  | 54 | 4.68 | 0.737 | – | |  |
| Total | – | – | – | 201/72/272 | 0.92 | 0.0213 |  | 0.961 | 0.0392 |  | 220 | 31.42 | 0.885 | – | |  |

Lat, latitude*;* long, longitude; alt, altitude (m a.s.l.); *n*, sample size for *Tpi*/ITS/nSSR analyses; *h*, haplotype diversity; *π*, nucleotide diversity; *N*_A_, total number of detected alleles; *R*_S_, allelic richness; *H*_S_, expected within-population (gene) diversity; *F*_IS_, inbreeding coefficient. Probabilities of significant inbreeding test (*F*_IS_) are marked with an asterisk.
