# Supplementary material for: A strong ‘filter’ effect of the East China Sea land bridge for East Asia’s temperate plant species: inferences from molecular phylogeography and ecological niche modelling of Platycrater arguta (Hydrangeaceae)
Source: BMC Evol Biol. 2014 Mar 4;14:41. doi: 10.1186/1471-2148-14-41 (PMC4015774; doi:10.1186/1471-2148-14-41)
Supplement: Additional file 2: Table S2 — Characteristics of 7 microsatellite loci developed for Platycrater arguta. Shown for each locus are the locus name, the forward (F) and reverse (R) primer sequence, repeat motifs, the optimized annealing temperature (Ta), allele size ranges, the GenBank accession number. [file 1471-2148-14-41-S2.docx]

**Additional file 2: Table S2.** Characteristics of 7 microsatellite loci developed for *Platycrater arguta*. Shown for each locus are the locus name, the forward (F) and reverse (R) primer sequence, repeat motifs, the optimized annealing temperature (*T*a), allele size ranges, the GenBank accession number.

| Locus | Primer sequence (5'–3') | Repeat motif | *T*a (°C) | Size Range (bp) | Genbank Accession No. |
| --- | --- | --- | --- | --- | --- |
| Pa1 (HEX) | F: GCTGGATAAGCTGTGAAT | (AG)_21_ | 54 | 143–185 | JN383518 |
|  | R: ACTTTGGAACAGATTGAA |  |  |  |  |
| Pa2 (HEX) | F: AGTATAGAGCTTACCTCA | (AG)_10_ | 54 | 160–226 | JN383519 |
|  | R: TATGTAGAAAGTTGTTAGT |  |  |  |  |
|  | R: TGTGGAGGCAACATAAAA |  |  |  |  |
| Pa3 | F: TTCCGAACAAACGAAGAT | (AG)_15_ | 54 | 176–200 | JN383520 |
|  | R: TGTGGAGGCAACATAAAA |  |  |  |  |
| Pa5 (HEX) | F: (AC)_6_(AG)_5_ | (AC)_6_(AG)_9_ | 54 | 183–225 | JN383521 |
|  | R: AATGGCGGTTTGGGTGGC |  |  |  |  |
| Pa6 (HEX) | F: (AC)_6_(AG)_5_ | (AC)_6_(AG)_5_ | 54 | 240–258 | JN383522 |
|  | R: TAGGGTTCTTGGATAGTGGA |  |  |  |  |
| Pa7 (FAM) | F: (AC)_6_(AG)_5_ | (AC)_5_(AG)_7_ | 54 | 154–164 | JN383523 |
|  | R: GGTCAGCAGGATGGAGCA |  |  |  |  |
| Pa8 (FAM) | F: (TC)_6_(AC)_5_ | (TC)_6_(AC)_9_ | 54 | 110–132 | JN383524 |
|  | R: GGAACAAACGAGCCGAAA |  |  |  |  |
