# Supplementary material for: A strong ‘filter’ effect of the East China Sea land bridge for East Asia’s temperate plant species: inferences from molecular phylogeography and ecological niche modelling of Platycrater arguta (Hydrangeaceae)
Source: BMC Evol Biol. 2014 Mar 4;14:41. doi: 10.1186/1471-2148-14-41 (PMC4015774; doi:10.1186/1471-2148-14-41)
Supplement: Additional file 3: Table S3 — ITS sequence polymorphism detected in Platycrater at ITS1 and ITS2 (the 5.8S excluded) regions and identifying 33 haplotypes (H1−H33). A dash (−) denotes a single nucleotide indel. Note that poly-A or poly-T stretches were excluded from analysis. [file 1471-2148-14-41-S3.docx]

**Additional file 3: Table S3.** ITS sequence polymorphism detected in *Platycrater* at ITS1 and ITS2 (the 5.8S excluded) regions and identifying 33 haplotypes (H1−H33). A dash (-) denotes a single nucleotide indel. Note that poly-A or poly-T stretches were excluded from analysis.

| Haplotype | Nucleotide position | | | | | | | | | | | | | | | | | | | | | | | | | | | | | | | | | | | | | | | | |
| --- | --- | --- | --- | --- | --- | --- | --- | --- | --- | --- | --- | --- | --- | --- | --- | --- | --- | --- | --- | --- | --- | --- | --- | --- | --- | --- | --- | --- | --- | --- | --- | --- | --- | --- | --- | --- | --- | --- | --- | --- | --- |
|  | ITS1 | | | | | | | | | | | | | | | | | | | | | | | | | | | | | | | | | | | | | | | | |
|  | 0 | 0 | 0 | 0 | 0 | 0 | 0 | 0 | 0 | 0 | 0 | 0 | 0 | 0 | 0 | 0 | 0 | 0 | 0 | 0 | 0 | 0 | 0 | 0 | 0 | 0 | 0 | 0 | 1 | 1 | 1 | 1 | 1 | 1 | 1 | 1 | 1 | 1 | 1 | 1 | 2 |
|  | 0 | 0 | 1 | 1 | 1 | 1 | 1 | 2 | 2 | 3 | 3 | 3 | 3 | 3 | 3 | 5 | 5 | 5 | 5 | 6 | 6 | 6 | 7 | 7 | 7 | 8 | 9 | 9 | 1 | 2 | 2 | 4 | 5 | 6 | 7 | 7 | 7 | 8 | 8 | 9 | 4 |
|  | 8 | 9 | 1 | 2 | 3 | 4 | 7 | 3 | 7 | 0 | 1 | 2 | 3 | 6 | 9 | 0 | 2 | 7 | 8 | 1 | 3 | 4 | 4 | 8 | 9 | 3 | 0 | 8 | 1 | 1 | 8 | 9 | 1 | 2 | 4 | 5 | 7 | 3 | 5 | 4 | 1 |
| H1 | C | A | A | A | G | A | A | A | G | T | T | A | G | C | C | G | A | G | G | G | G | A | A | T | G | G | A | T | C | T | A | G | A | T | G | G | T | C | C | A | A |
| H2 | . | . | . | . | . | . | . | . | . | . | C | . | . | . | . | . | . | . | . | . | . | . | . | . | . | . | . | . | . | . | . | . | . | . | . | . | . | . | . | . | . |
| H3 | . | . | . | . | . | . | . | . | . | . | C | . | C | . | . | . | . | . | . | . | . | . | . | . | . | . | . | . | . | . | . | . | . | . | . | . | . | . | . | . | . |
| H4 | . | . | . | . | . | . | . | . | . | . | . | . | . | . | . | . | . | . | . | . | . | . | . | . | . | . | . | . | . | . | . | . | . | . | . | . | . | . | . | . | . |
| H5 | . | . | . | . | . | . | . | . | . | . | . | . | . | . | . | . | . | . | . | . | . | . | . | . | . | . | . | . | . | . | . | . | . | . | . | . | . | . | . | . | . |
| H6 | . | . | . | . | . | . | . | . | . | . | . | . | . | . | . | . | . | . | . | . | . | . | . | . | . | . | . | . | . | . | . | . | . | . | . | . | . | . | . | . | . |
| H7 | . | . | T | . | . | . | . | . | . | . | . | . | . | . | . | . | . | . | . | . | . | . | . | . | . | . | . | . | . | . | . | . | . | . | . | . | . | . | . | . | . |
| H8 | . | . | . | . | . | . | . | . | . | . | . | . | . | . | . | . | . | . | . | . | . | . | . | . | . | . | . | . | . | . | . | . | . | . | . | . | . | . | . | . | . |
| H9 | . | . | G | . | . | . | . | . | . | . | . | . | . | . | . | . | . | . | . | . | . | . | . | . | . | . | . | . | . | . | . | . | . | . | . | . | . | . | . | . | . |
| H10 | G | . | G | G | G | C | . | G | . | G | . | G | . | T | . | . | . | . | . | A | . | . | G | A | . | . | . | . | T | . | . | . | G | . | A | A | C | A | T | G | G |
| H11 | G | . | G | G | G | C | . | G | . | G | . | G | . | T | . | . | . | . | . | A | . | . | G | A | . | . | . | . | T | . | . | . | G | . | A | A | C | . | T | G | G |
| H12 | G | . | G | . | . | . | G | . | . | G | . | . | . | . | . | . | G | A | . | A | . | . | . | . | . | . | . | . | . | C | G | . | . | . | . | A | C | . | T | . | . |
| H13 | . | . | . | . | . | . | . | . | . | . | . | . | . | . | . | . | . | . | . | . | . | . | . | . | . | . | . | . | . | . | . | . | . | . | . | . | . | . | . | . | . |
| H14 | G | . | G | . | . | . | . | . | . | G | . | . | . | . | . | T | G | A | - | A | A | . | . | . | . | . | . | . | . | C | G | T | . | . | . | A | C | . | T | . | . |
| H15 | G | . | . | . | . | . | G | . | . | G | . | . | . | . | . | . | G | A | . | A | . | . | . | . | . | . | . | . | . | C | G | . | . | . | . | A | C | . | T | G | . |
| H16 | G | . | . | . | . | . | G | . | . | G | . | . | . | . | . | . | G | A | . | A | . | . | . | . | . | . | . | . | . | C | G | . | . | . | . | A | C | . | T | G | . |
| H17 | G | . | G | . | - | . | G | . | . | G | . | . | . | . | . | . | G | A | . | A | . | . | . | . | . | . | . | . | . | C | G | . | . | . | . | A | C | . | T | G | . |
| H18 | G | . | . | . | . | . | G | . | . | G | . | . | . | . | . | . | G | A | . | A | . | . | . | . | . | . | . | . | . | C | G | . | . | . | . | A | C | . | T | G | . |
| H19 | G | . | . | . | . | . | G | . | . | G | . | . | . | . | . | . | G | A | . | A | . | . | . | . | . | . | . | . | . | C | G | . | . | . | . | A | C | . | T | G | . |
| H20 | G | . | . | . | . | . | G | . | . | G | . | . | . | . | . | . | G | . | . | A | . | . | . | . | . | . | . | . | . | C | G | . | . | . | . | A | C | . | T | G | . |
| H21 | G | . | . | . | . | . | G | . | . | G | . | . | . | . | . | . | G | A | . | A | . | . | . | . | . | . | . | . | . | C | G | . | . | . | . | A | C | . | T | G | . |
| H22 | G | . | . | . | . | . | . | . | A | G | . | . | C | . | . | . | G | A | . | A | . | . | . | . | . | . | . | . | . | C | G | . | . | . | . | A | C | . | T | G | . |
| H23 | G | . | G | - | - | - | . | G | . | G | . | G | . | T | . | . | . | . | . | A | . | . | G | C | T | A | . | . | T | C | . | . | G | . | A | A | . | . | . | G | G |
| H24 | G | . | G | - | - | - | . | G | . | G | . | G | . | T | . | . | . | . | . | A | . | . | G | C | T | A | . | . | T | C | . | . | G | . | A | A | . | . | . | G | G |
| H25 | G | . | . | . | . | . | G | . | . | G | . | G | . | . | . | . | G | A | . | A | . | . | . | . | . | . | . | . | . | C | G | . | . | . | . | A | C | . | T | G | . |
| H26 | G | . | G | - | - | - | . | G | . | G | . | G | . | T | . | . | . | . | . | A | . | . | G | C | T | A | . | . | T | C | . | . | G | . | A | A | . | . | . | G | G |
| H27 | G | . | G | - | - | - | . | G | . | G | . | G | . | T | . | . | . | . | . | A | . | . | G | C | T | A | . | . | T | C | . | . | G | . | A | A | . | . | . | G | G |
| H28 | G | . | G | - | - | - | . | G | . | G | . | G | . | T | A | . | . | . | . | A | . | G | G | C | . | . | . | C | T | . | . | . | . | C | A | A | . | . | T | G | G |
| H29 | G | . | G | - | - | - | . | G | . | G | . | G | . | T | . | . | . | . | . | A | . | . | G | C | T | A | T | . | T | C | . | . | G | . | A | A | . | . | . | G | G |
| H30 | G | . | G | - | - | - | . | G | . | G | . | G | . | T | . | . | . | . | . | A | . | . | G | C | T | A | T | . | T | C | . | . | G | . | A | A | . | . | . | G | G |
| H31 | G | . | . | - | - | - | . | G | . | G | . | G | . | T | . | . | . | . | . | A | . | . | G | A | . | A | . | . | T | . | . | . | G | . | A | A | C | . | T | G | G |
| H32 | G | . | G | G | G | C | . | G | . | G | . | G | . | T | . | . | . | . | . | A | . | . | G | A | . | . | . | . | T | . | . | . | G | . | A | A | C | . | T | G | G |
| H33 | G | G | G | - | - | - | . | G | . | G | . | G | . | T | . | . | . | . | . | A | . | . | G | A | . | . | . | . | T | . | . | . | G | . | A | A | C | . | T | G | G |

| Nucleotide position | | | | | | | | | | | | | | | | | | | | | | | | | | | | | | | | | | | | | | | | | | |
| --- | --- | --- | --- | --- | --- | --- | --- | --- | --- | --- | --- | --- | --- | --- | --- | --- | --- | --- | --- | --- | --- | --- | --- | --- | --- | --- | --- | --- | --- | --- | --- | --- | --- | --- | --- | --- | --- | --- | --- | --- | --- | --- |
| ITS2 | | | | | | | | | | | | | | | | | | | | | | | | | | | | | | | | | | | | | | | | | | |
| 3 | 3 | 3 | 3 | 4 | 4 | 4 | 4 | 4 | 4 | 4 | 4 | 4 | 4 | 4 | 4 | 4 | 4 | 4 | 4 | 4 | 5 | 5 | 5 | 5 | 5 | 5 | 5 | 5 | 5 | 5 | 5 | 5 | 5 | 5 | 5 | 5 | 5 | 5 | 5 | 5 | 6 |  |
| 7 | 8 | 8 | 9 | 0 | 0 | 0 | 0 | 0 | 1 | 1 | 2 | 4 | 5 | 5 | 7 | 7 | 8 | 9 | 9 | 9 | 0 | 0 | 1 | 1 | 2 | 2 | 2 | 3 | 3 | 3 | 4 | 4 | 4 | 5 | 5 | 6 | 6 | 7 | 7 | 7 | 0 |  |
| 9 | 0 | 1 | 6 | 1 | 2 | 3 | 8 | 9 | 2 | 4 | 4 | 8 | 1 | 4 | 0 | 2 | 5 | 1 | 4 | 7 | 7 | 9 | 7 | 9 | 4 | 7 | 9 | 0 | 4 | 7 | 2 | 4 | 8 | 0 | 6 | 2 | 8 | 3 | 5 | 6 | 3 |  |
| C | T | T | C | A | G | A | G | G | G | A | G | A | T | T | T | T | C | A | G | A | A | T | G | G | G | C | G | A | G | T | A | G | G | T | T | T | A | A | A | A | C |  |
| . | . | . | . | . | . | . | . | . | . | . | . | . | . | . | . | . | . | . | . | . | . | C | . | . | . | . | . | . | . | . | . | . | . | . | . | . | . | . | . | . | . |  |
| . | . | . | . | . | . | . | . | . | . | . | . | . | . | . | . | . | . | G | . | . | . | . | . | . | . | . | . | . | . | . | . | . | . | . | C | . | . | . | . | . | . |  |
| . | . | . | . | . | . | . | . | . | . | . | . | . | . | . | . | . | . | G | . | . | . | . | . | . | . | . | . | . | . | . | . | . | . | . | . | . | . | . | . | . | . |  |
| . | . | . | . | . | . | . | . | . | . | . | . | . | . | . | . | . | . | . | . | . | . | C | . | . | . | . | . | . | . | . | . | . | . | . | C | . | G | . | . | . | . |  |
| . | . | . | . | . | . | . | . | . | . | . | . | . | . | . | . | . | . | G | . | . | . | . | . | . | . | . | . | . | . | . | . | . | . | . | C | . | G | . | . | . | . |  |
| . | . | . | . | . | . | G | . | . | . | . | . | . | . | . | . | . | . | . | . | . | . | . | . | . | . | . | . | . | . | . | . | . | . | . | . | . | . | . | . | . | . |  |
| . | . | . | . | . | . | G | . | . | . | . | . | . | . | . | . | . | . | . | . | . | . | . | . | . | . | . | . | . | . | . | . | . | . | . | . | . | . | . | . | . | . |  |
| . | . | . | . | . | . | G | . | . | . | . | . | . | . | . | . | . | . | . | . | . | . | C | . | . | . | . | . | . | . | . | . | . | . | . | . | . | . | . | . | . | . |  |
| T | . | C | A | . | A | . | . | . | A | . | . | . | . | . | C | C | A | . | . | G | G | . | . | . | A | . | . | G | . | A | . | . | . | C | . | . | . | . | . | . | . |  |
| T | . | C | A | . | A | . | . | . | A | . | . | . | . | . | C | C | A | . | . | G | G | . | . | . | A | . | . | G | . | A | . | . | . | C | . | . | . | . | . | . | . |  |
| . | . | . | . | . | . | . | . | . | . | . | A | . | C | . | . | . | . | . | . | . | . | . | . | T | . | . | . | . | . | . | G | . | . | . | C | . | . | . | . | . | . |  |
| . | . | . | . | . | . | . | . | . | . | . | . | . | . | . | . | . | . | G | . | . | . | . | . | . | . | . | . | . | . | . | . | . | . | . | C | . | . | . | . | . | . |  |
| . | . | . | . | . | . | . | . | . | . | . | A | . | C | . | . | . | . | . | . | . | . | . | . | . | . | . | C | . | . | . | G | . | . | . | . | . | . | . | . | . | . |  |
| . | . | . | . | . | . | . | . | . | . | . | . | . | C | . | . | . | . | . | . | . | . | . | C | . | . | . | T | T | . | . | G | . | . | . | . | . | . | . | . | . | T |  |
| . | . | . | . | . | . | . | . | . | . | . | . | . | C | . | . | . | . | . | . | . | . | . | . | . | . | . | . | . | . | . | G | . | . | . | . | . | . | . | . | . | . |  |
| . | . | . | . | . | . | . | . | . | . | . | . | . | C | . | . | . | . | . | . | . | . | . | . | . | . | . | . | . | . | . | G | . | . | . | . | . | . | . | . | . | . |  |
| . | C | . | . | . | . | . | . | . | . | . | . | . | C | . | . | . | . | . | . | . | . | . | . | . | . | . | . | . | . | . | G | . | . | . | . | . | . | . | . | . | . |  |
| . | C | . | . | . | . | . | . | . | . | . | . | . | C | . | . | . | . | . | . | . | . | . | . | . | . | . | . | . | A | . | G | . | . | . | . | . | . | . | . | . | . |  |
| . | C | . | . | . | . | . | . | . | . | . | . | . | C | . | . | . | . | . | . | . | . | . | . | . | . | . | . | . | A | . | G | . | . | . | . | . | . | . | . | . | . |  |
| . | C | . | . | . | . | . | T | T | . | . | . | . | C | . | . | . | . | . | . | . | . | . | . | . | . | . | . | . | A | . | G | . | . | . | . | . | . | . | . | . | . |  |
| . | C | . | . | . | . | . | . | . | . | . | . | . | C | . | . | . | . | . | . | . | . | . | . | . | . | . | . | . | . | . | G | . | . | . | . | . | . | . | . | . | . |  |
| T | C | C | A | . | A | . | . | . | A | . | . | . | . | . | C | . | A | . | A | G | G | . | . | . | A | . | . | G | . | . | G | . | . | C | . | . | G | . | . | G | . |  |
| T | C | C | A | . | A | . | . | . | A | . | . | . | . | . | C | . | A | . | A | G | G | . | . | . | A | . | . | G | . | . | . | . | . | C | . | . | G | . | . | G | . |  |
| . | C | . | . | . | . | . | . | . | . | . | . | . | C | . | . | . | . | . | . | . | . | . | . | . | . | . | . | . | A | . | G | . | . | . | . | . | . | . | . | . | . |  |
| T | C | C | A | . | A | . | . | . | A | . | . | . | . | . | C | . | A | . | A | G | G | C | . | . | A | . | . | G | . | . | . | . | . | C | . | . | G | . | . | G | . |  |
| T | C | C | A | . | A | . | . | . | A | . | . | . | . | . | C | . | A | . | A | G | G | C | . | . | A | . | . | G | . | . | G | T | C | C | . | . | G | . | . | G | . |  |
| T | . | C | . | G | A | G | . | . | A | G | . | G | . | . | C | . | A | . | A | G | G | C | . | . | . | . | . | G | . | . | . | . | . | C | . | C | G | G | . | G | . |  |
| T | C | C | A | . | A | . | . | . | A | . | . | . | . | . | C | . | A | . | A | G | G | . | . | . | A | . | . | G | . | . | . | . | . | C | . | . | G | . | . | G | . |  |
| T | C | C | A | . | A | . | . | . | A | . | . | . | . | . | C | . | A | . | A | G | G | . | . | . | A | G | . | G | . | . | G | . | . | C | . | . | G | . | . | G | . |  |
| T | . | C | A | . | A | . | . | . | A | . | . | . | . | . | C | . | A | . | . | G | G | . | . | . | A | . | . | G | . | . | . | . | . | C | . | . | . | . | . | . | . |  |
| T | . | C | A | . | A | . | . | . | A | . | . | . | . | . | C | . | A | . | . | G | G | . | . | . | A | . | . | G | . | . | . | . | . | C | . | . | . | . | . | . | . |  |
| T | . | C | A | . | A | . | . | . | A | . | . | . | . | C | C | . | A | . | . | G | G | . | . | . | . | . | . | G | . | . | . | . | . | C | . | . | . | . | G | . | . |  |
