# Supplementary material for: A strong ‘filter’ effect of the East China Sea land bridge for East Asia’s temperate plant species: inferences from molecular phylogeography and ecological niche modelling of Platycrater arguta (Hydrangeaceae)
Source: BMC Evol Biol. 2014 Mar 4;14:41. doi: 10.1186/1471-2148-14-41 (PMC4015774; doi:10.1186/1471-2148-14-41)
Supplement: Additional file 4: Table S4 — The sequence polymorphism detected in Platycrater at Tpi gene region. The 31 haplotypes were denoting as T1−T 31. A dash (−) denotes a single nucleotide indel. [file 1471-2148-14-41-S4.docx]

**Additional file 4: Table S4.** The sequence polymorphism detected in *Platycrater* at *Tpi* gene region. The 31 haplotypes were denoting as T1−T 31. A dash (-) denotes a single nucleotide indel.

| Haplotype | Nucleotide position | | | | | | | | | | | | | | | | | | | | | | | | | | | | | | | | | | | | | | | | | | |
| --- | --- | --- | --- | --- | --- | --- | --- | --- | --- | --- | --- | --- | --- | --- | --- | --- | --- | --- | --- | --- | --- | --- | --- | --- | --- | --- | --- | --- | --- | --- | --- | --- | --- | --- | --- | --- | --- | --- | --- | --- | --- | --- | --- |
|  | 0 | 0 | 0 | 0 | 0 | 0 | 0 | 0 | 0 | 0 | 0 | 0 | 0 | 0 | 0 | 1 | 1 | 1 | 1 | 1 | 1 | 1 | 1 | 1 | 1 | 2 | 2 | 2 | 2 | 2 | 2 | 2 | 2 | 2 | 2 | 2 | 2 | 2 | 2 | 3 | 3 | 3 |  |
|  | 0 | 1 | 2 | 2 | 3 | 4 | 5 | 5 | 5 | 6 | 6 | 7 | 7 | 8 | 8 | 0 | 0 | 1 | 1 | 1 | 1 | 2 | 3 | 6 | 7 | 1 | 2 | 3 | 3 | 3 | 3 | 3 | 3 | 5 | 5 | 6 | 7 | 7 | 8 | 0 | 1 | 3 |  |
|  | 6 | 3 | 0 | 3 | 2 | 5 | 2 | 6 | 7 | 0 | 6 | 5 | 9 | 2 | 8 | 0 | 7 | 0 | 2 | 4 | 9 | 9 | 0 | 8 | 4 | 1 | 9 | 1 | 5 | 6 | 7 | 8 | 9 | 6 | 8 | 8 | 4 | 5 | 0 | 5 | 1 | 9 |  |
| T1 | A | A | C | T | G | T | G | T | T | C | C | T | A | T | G | C | A | A | T | G | A | T | C | A | G | T | G | T | C | G | T | T | A | T | A | T | T | C | A | C | A | A |  |
| T2 | . | C | . | . | . | . | . | . | . | . | . | . | . | . | . | . | . | . | . | . | . | . | T | . | A | G | . | . | . | . | . | . | . | . | . | . | . | . | . | T | . | . |  |
| T3 | . | . | . | . | . | . | . | . | . | . | . | . | . | . | . | . | . | . | . | . | . | . | . | . | . | G | . | . | . | . | . | . | . | . | . | . | . | . | . | T | . | . |  |
| T4 | . | C | . | . | . | . | . | . | . | . | . | . | . | . | . | . | . | . | . | . | . | . | . | . | A | G | . | . | . | . | . | . | . | . | . | . | . | . | . | T | . | . |  |
| T5 | . | C | . | A | . | . | . | . | C | . | . | C | G | . | . | . | . | . | . | . | . | A | . | . | A | A | . | . | - | - | - | - | - | . | . | . | A | T | . | T | . | . |  |
| T6 | . | C | . | . | . | . | . | C | C | . | . | C | G | . | . | . | . | . | . | . | . | A | . | . | A | G | T | . | . | . | A | . | . | . | . | . | A | T | . | T | . | . |  |
| T7 | . | C | . | . | . | C | . | C | C | . | . | C | G | . | . | G | . | . | C | . | T | A | . | . | A | G | T | . | . | . | A | . | . | . | . | . | A | T | . | T | T | . |  |
| T8 | . | C | . | . | A | . | A | C | C | . | . | C | G | . | . | G | . | . | C | . | . | A | . | . | A | G | T | . | . | . | A | . | . | . | . | . | A | T | . | T | . | . |  |
| T9 | . | C | . | . | A | . | A | C | C | . | . | C | G | . | . | G | . | . | C | . | . | A | . | . | A | G | T | . | . | . | A | . | . | . | . | C | A | T | . | T | . | . |  |
| T10 | . | C | . | . | . | . | . | C | C | . | . | C | G | . | . | . | . | . | . | . | . | A | . | . | A | G | T | . | . | . | G | . | . | . | . | . | A | T | . | T | . | . |  |
| T11 | . | C | . | . | . | . | . | . | . | . | . | . | . | . | . | . | G | . | . | . | . | . | . | . | A | G | . | . | . | . | . | . | . | . | . | . | . | T | . | T | . | . |  |
| T12 | . | C | . | . | . | . | . | . | . | . | . | . | . | . | . | . | G | . | . | . | . | . | T | . | A | G | . | . | . | . | . | . | . | . | . | . | . | T | . | T | . | . |  |
| T13 | . | C | . | . | . | . | . | . | . | . | . | . | . | . | . | . | . | T | . | . | . | . | . | . | A | G | . | . | . | . | . | . | . | . | . | . | . | . | . | T | . | . |  |
| T14 | . | C | . | . | . | . | . | . | . | . | . | . | . | . | . | . | G | . | . | . | . | . | . | . | A | G | . | . | . | . | . | . | . | . | . | . | . | T | C | T | . | . |  |
| T15 | . | C | T | A | . | . | . | . | C | . | . | C | G | . | . | . | . | . | . | . | . | A | . | . | A | A | . | . | - | - | - | - | - | . | . | . | A | T | . | T | . | . |  |
| T16 | . | C | . | . | . | . | . | . | . | . | . | . | . | . | . | . | . | T | . | . | . | . | . | T | A | G | . | . | . | . | . | . | . | . | . | . | . | . | . | T | . | . |  |
| T17 | . | C | . | . | . | . | . | C | C | T | . | C | G | . | . | G | . | . | C | . | . | A | . | . | A | G | T | . | . | . | A | . | . | . | . | . | A | T | . | T | T | . |  |
| T18 | . | C | . | . | . | . | . | C | C | . | . | C | G | . | . | . | . | . | . | . | . | A | . | . | A | G | T | . | . | . | A | . | . | A | . | . | A | T | . | T | T | . |  |
| T19 | . | C | . | . | . | . | . | . | . | . | . | . | . | . | . | . | . | T | . | . | . | . | . | . | A | G | A | . | . | . | . | . | . | . | . | . | . | . | . | T | . | . |  |
| T20 | T | C | . | . | . | . | . | C | C | . | . | C | G | . | . | G | . | . | C | . | . | A | . | . | A | G | T | . | . | . | A | . | . | . | . | . | A | T | . | T | . | . |  |
| T21 | . | C | . | . | . | . | . | C | C | . | . | C | G | . | . | G | . | . | C | . | . | A | . | . | A | G | T | . | . | . | A | . | . | . | . | . | A | T | . | T | . | . |  |
| T22 | . | C | . | . | . | . | . | C | C | . | . | C | G | . | . | G | . | . | C | . | . | A | . | . | A | G | T | . | . | . | A | . | . | . | . | . | A | T | . | T | . | - |  |
| T23 | . | C | . | . | . | . | . | C | C | . | . | C | G | . | A | G | . | . | C | . | . | A | . | . | A | G | T | . | . | . | A | . | . | . | . | . | A | T | . | T | . | . |  |
| T24 | . | C | . | . | . | . | . | C | C | . | . | C | G | . | . | G | . | . | C | . | . | A | . | . | A | G | T | C | . | . | A | . | . | . | G | . | A | T | . | T | T | . |  |
| T25 | T | C | . | . | . | . | . | C | C | . | . | C | G | . | . | G | . | . | C | . | . | A | . | . | A | G | T | . | . | . | A | . | . | . | . | . | A | T | . | T | . | C |  |
| T26 | . | C | . | . | . | . | . | C | C | . | . | C | G | . | . | G | . | . | C | . | . | A | . | . | A | G | T | C | . | . | A | . | . | . | G | . | A | T | . | T | T | - |  |
| T27 | . | C | . | . | . | . | . | C | C | . | . | C | G | . | . | G | . | . | C | A | . | A | . | . | A | G | T | C | . | . | A | . | . | . | G | . | A | T | . | T | T | . |  |
| T28 | . | C | . | . | . | . | . | C | C | . | T | C | G | . | . | G | . | . | C | . | . | A | . | . | A | G | T | . | . | . | A | . | . | . | . | . | A | T | . | T | . | . |  |
| T29 | . | C | . | . | . | . | . | C | C | . | . | C | G | . | . | G | . | . | C | . | . | A | . | . | A | G | T | C | G | . | A | . | . | . | G | . | A | T | . | T | T | . |  |
| T30 | . | C | . | . | . | . | . | C | C | . | . | C | G | - | . | . | . | . | . | . | . | A | . | . | A | G | T | . | . | . | A | . | . | . | . | . | A | T | . | T | . | . |  |
| T31 | . | C | . | . | . | . | . | C | C | . | . | C | G | . | . | G | . | . | C | . | . | A | . | . | A | G | T | . | T | . | A | . | . | . | . | . | A | T | . | T | . | . |  |
