# Supplementary material for: A strong ‘filter’ effect of the East China Sea land bridge for East Asia’s temperate plant species: inferences from molecular phylogeography and ecological niche modelling of Platycrater arguta (Hydrangeaceae)
Source: BMC Evol Biol. 2014 Mar 4;14:41. doi: 10.1186/1471-2148-14-41 (PMC4015774; doi:10.1186/1471-2148-14-41)
Supplement: Additional file 5: Table S5 — Tests of neutrality and population growth for nDNA (ITS, Tpi) sequence variation in Platycrater arguta and each variety. [file 1471-2148-14-41-S5.docx]

**Additional file 5: Table S5.** Tests of neutrality and population growth for nDNA (ITS, *Tpi*) sequence variation in *Platycrater arguta* and each variety.

|  | ITS | | | | |  | *Tpi* | | | | |
| --- | --- | --- | --- | --- | --- | --- | --- | --- | --- | --- | --- |
| Species/variety (region) | Neutrality | |  | Population growth | |  | Neutrality | |  | Population growth | |
|  | *D* | *D** |  | *F*_S_ | *R*_2_ |  | *D* | *D** |  | *F*_S_ | *R*_2_ |
| *Platycrater arguta* (total) | -0.1192 | -0.0960 |  | -0.1020 | 0.0979** |  | -0.0614 | -0.0532 |  | -0.1376 | 0.1622** |
| var. *sinensis* (China) | -0.0861 | -0.0706 |  | -0.1280 | 0.1141** |  | -0.0641 | -0.0652 |  | -0.0920 | 0.0865** |
| var. *arguta* (Japan) | -0.0897 | -0.0929 |  | -0.0863 | 0.1117** |  | -0.0726 | -0.0812 |  | -0.0931 | 0.1030** |

Neutrality tests: *D*, Tajima’s *D*; *D**, Fu and Li's *D**. Population growth tests: *F*_S_, Fu’s *F*_S_; *R*_2_, Ramos-Onsins & Rozas’ *R*_2_. ** *P* < 0.01.
