# Supplementary material for: A strong ‘filter’ effect of the East China Sea land bridge for East Asia’s temperate plant species: inferences from molecular phylogeography and ecological niche modelling of Platycrater arguta (Hydrangeaceae)
Source: BMC Evol Biol. 2014 Mar 4;14:41. doi: 10.1186/1471-2148-14-41 (PMC4015774; doi:10.1186/1471-2148-14-41)
Supplement: Additional file 6: Table S6 — Mean estimates (± SE) of gene diversity within populations (hS), total gene diversity (hT), and population differentiation for unordered (GST) and ordered (NST) haplotypes of ITS and Tpi in Platycrater arguta and each variety. 1,000 random permutations were performed to test whether NST is significantly larger than GST. [file 1471-2148-14-41-S6.docx]

**Additional file 6: Table S6.** Mean estimates (± SE) of gene diversity within populations (*h*_S_), total gene diversity (*h*_T_), and population differentiation for unordered (*G*_ST_) and ordered (*N*_ST_) haplotypes of ITS and *Tpi* in *Platycrater arguta* and each variety. 1,000 random permutations were performed to test whether *N*_ST_ is significantly larger than *G*_ST._

| Species/variety (region) | *h*_S_ | *h*_T_ | *G*_ST_ | *N*_ST_ |
| --- | --- | --- | --- | --- |
| ITS |  |  |  |  |
| *Platycrater arguta* (total) | 0.461 (0.0937) | 0.990 (0.0169) | 0.534 (0.0939) | 0.934 (0.0209)^*^ |
| var. *sinensis* (China) | 0.424 (0.1217) | 0.955 (0.0525) | 0.556 (0.1233) | 0.751 (0.1452)^*^ |
| var. *arguta* (Japan) | 0.498 (0.1510) | 1.000 (0.0366) | 0.502 (0.1521) | 0.907 (0.0461)^*^ |
| *Tpi* |  |  |  |  |
| *Platycrater arguta* (total) | 0.513 (0.0806) | 0.957 (0.0346) | 0.464 (0.0750) | 0.812 (0.0473)^*^ |
| var. *sinensis* (China) | 0.591 (0.1059) | 0.970 (0.0275) | 0.391 (0.1157) | 0.458 (0.1244)^NS^ |
| var. *arguta* (Japan) | 0.435 (0.1222) | 0.842 (0.1179) | 0.483 (0.0828) | 0.775 (0.1465)^*^ |

* indicates that *N*_ST_ is significantly larger than *G*_ST_ (*P* < 0.01); NS, not significant.
