# Supplementary figures and images for: A strong ‘filter’ effect of the East China Sea land bridge for East Asia’s temperate plant species: inferences from molecular phylogeography and ecological niche modelling of Platycrater arguta (Hydrangeaceae)
Source: BMC Evol Biol. 2014 Mar 4;14:41. doi: 10.1186/1471-2148-14-41 (PMC4015774; doi:10.1186/1471-2148-14-41)

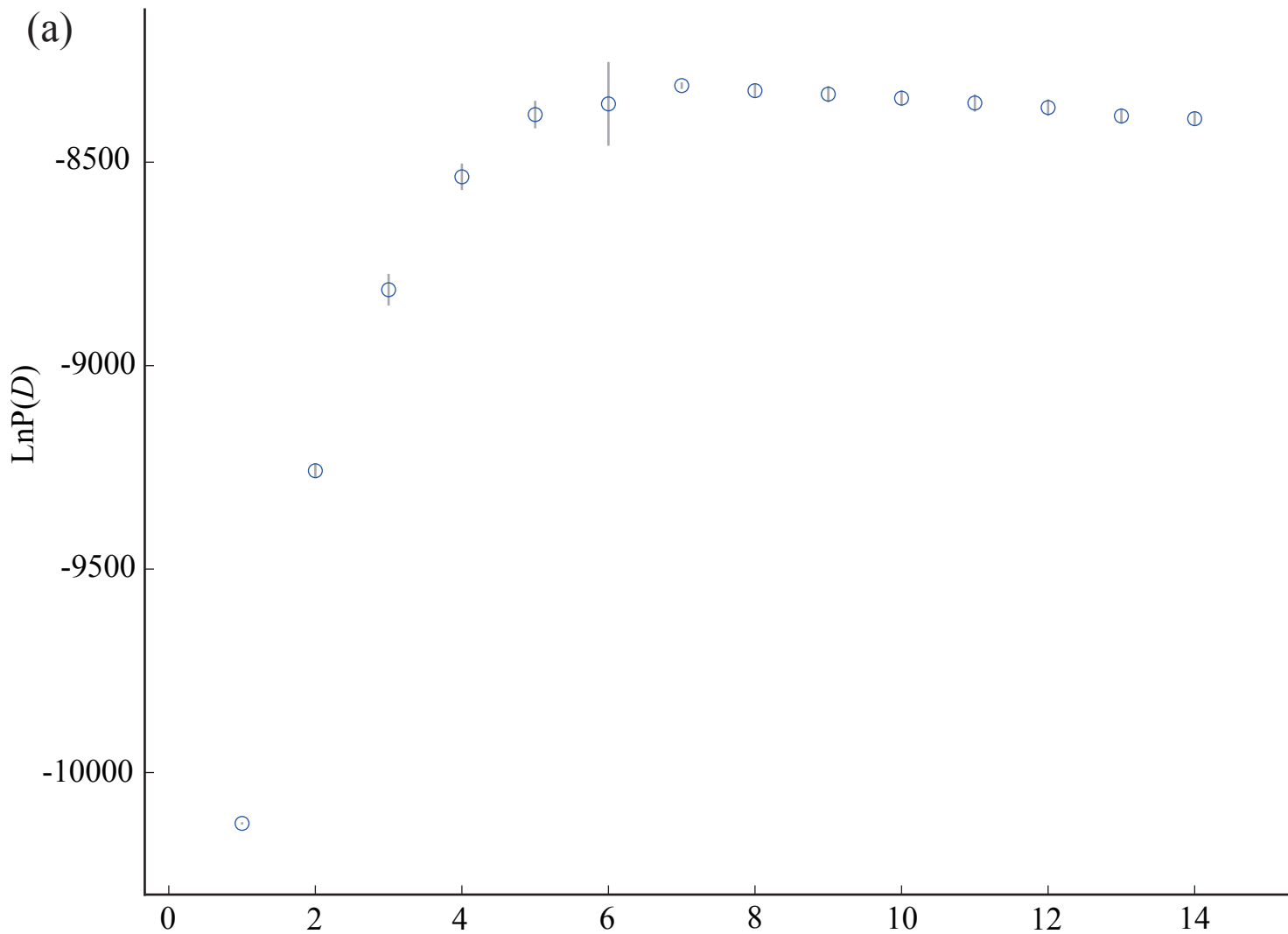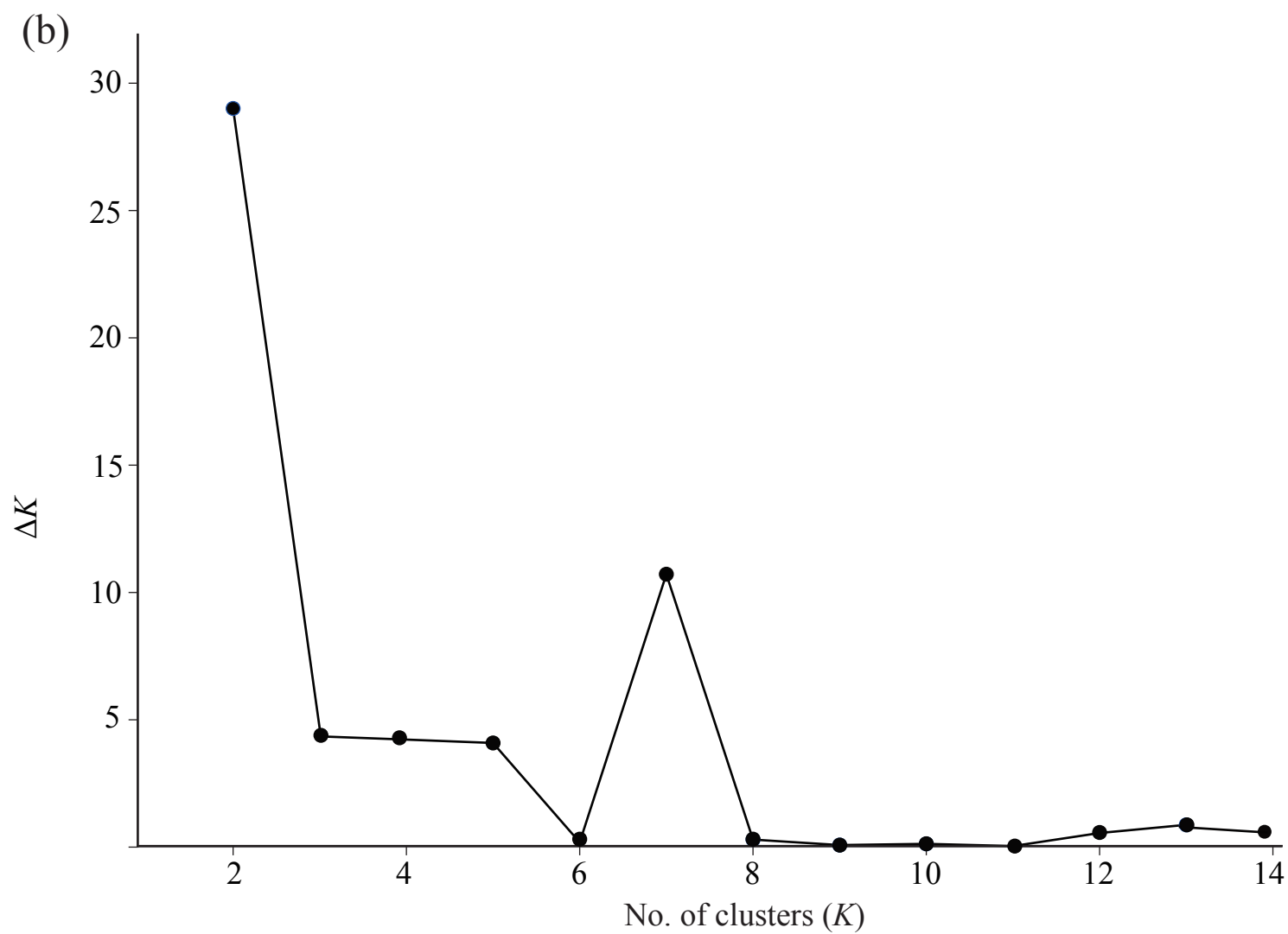

Supplement: Additional file 7: Figure S1 — (a) The posterior probability of the nSSR data on 14 Platycrater arguta populations given K clusters, obtained through 10 runs of the STRUCTURE algorithm (Falush et al. [53]). (b) The corresponding ΔK statistic (Evanno et al. [54]) showing peaks at K = 2 and K = 7, indicating that those are the best solutions for K given the data. [file 1471-2148-14-41-S7.pdf]

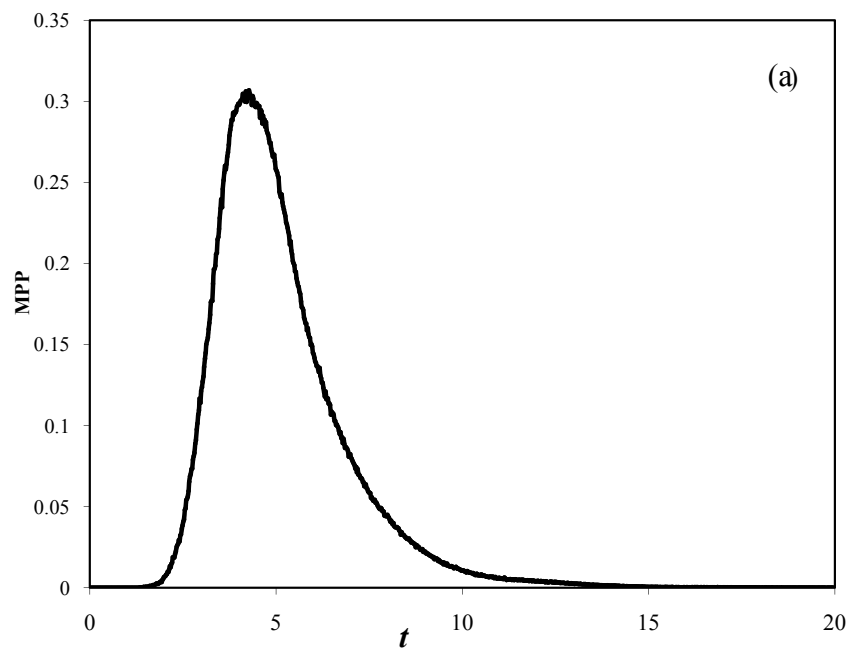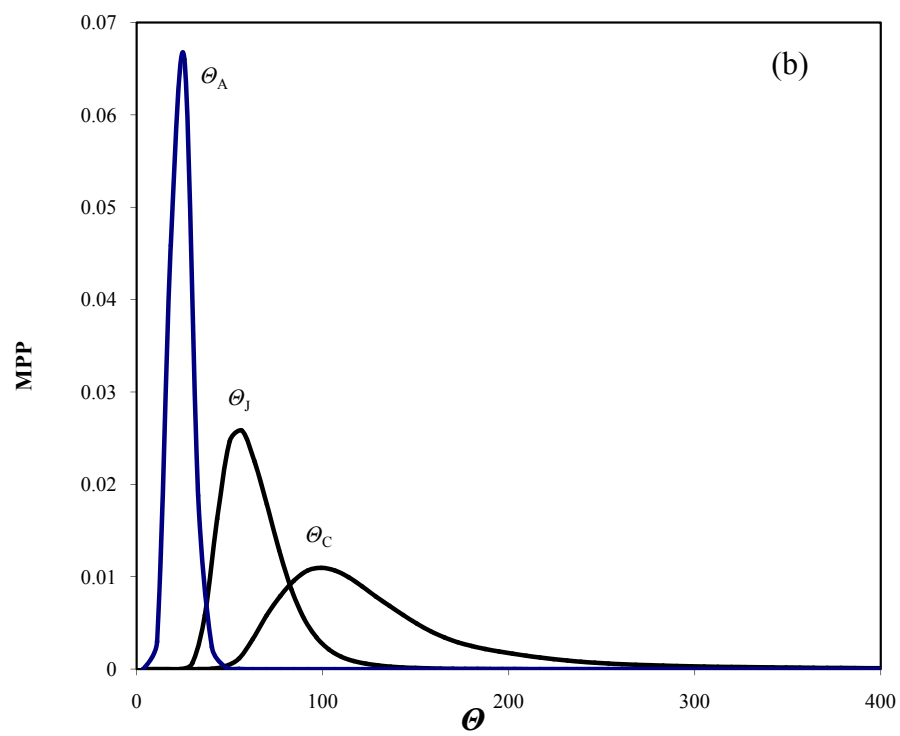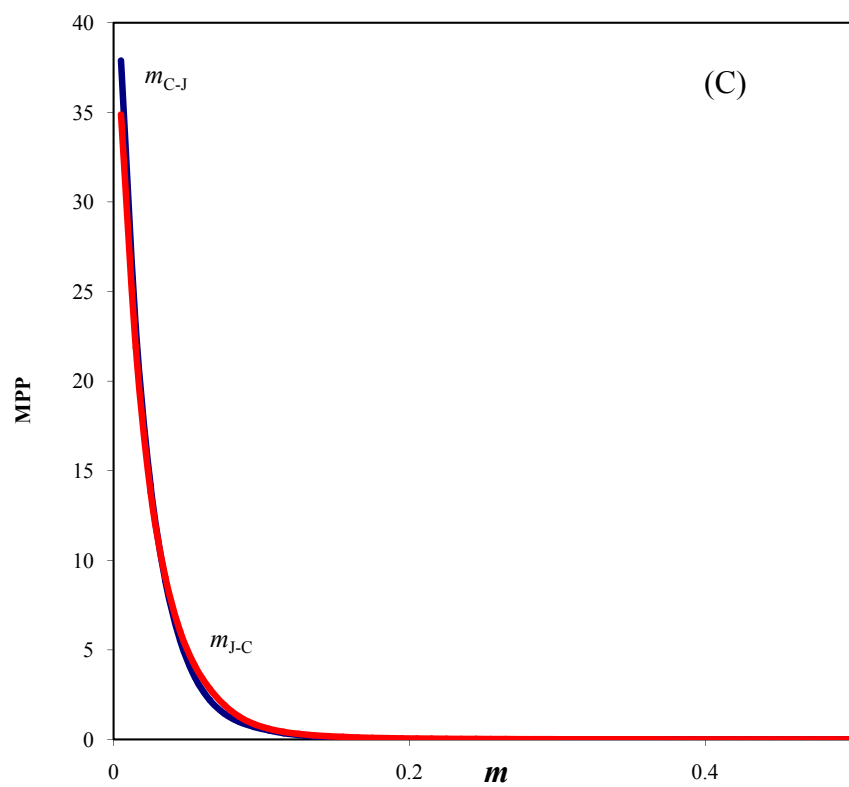

Supplement: Additional file 9: Figure S2 — Marginal posterior probability (MPP) distributions of six IMA-derived model parameters for Platycrater arguta var. sinensis (East China) and var. arguta (South Japan). (a) The time (t) since ancestral population splitting in mutational units. (b) The scaled effective population sizes (Θ) of var. sinensis (ΘC), var. arguta (ΘJ), and the ancestral population (ΘA). (c) The scaled migration rates forward in time from East China to South Japan (mC-J), and vice versa (mJ-C). [file 1471-2148-14-41-S9.pdf]

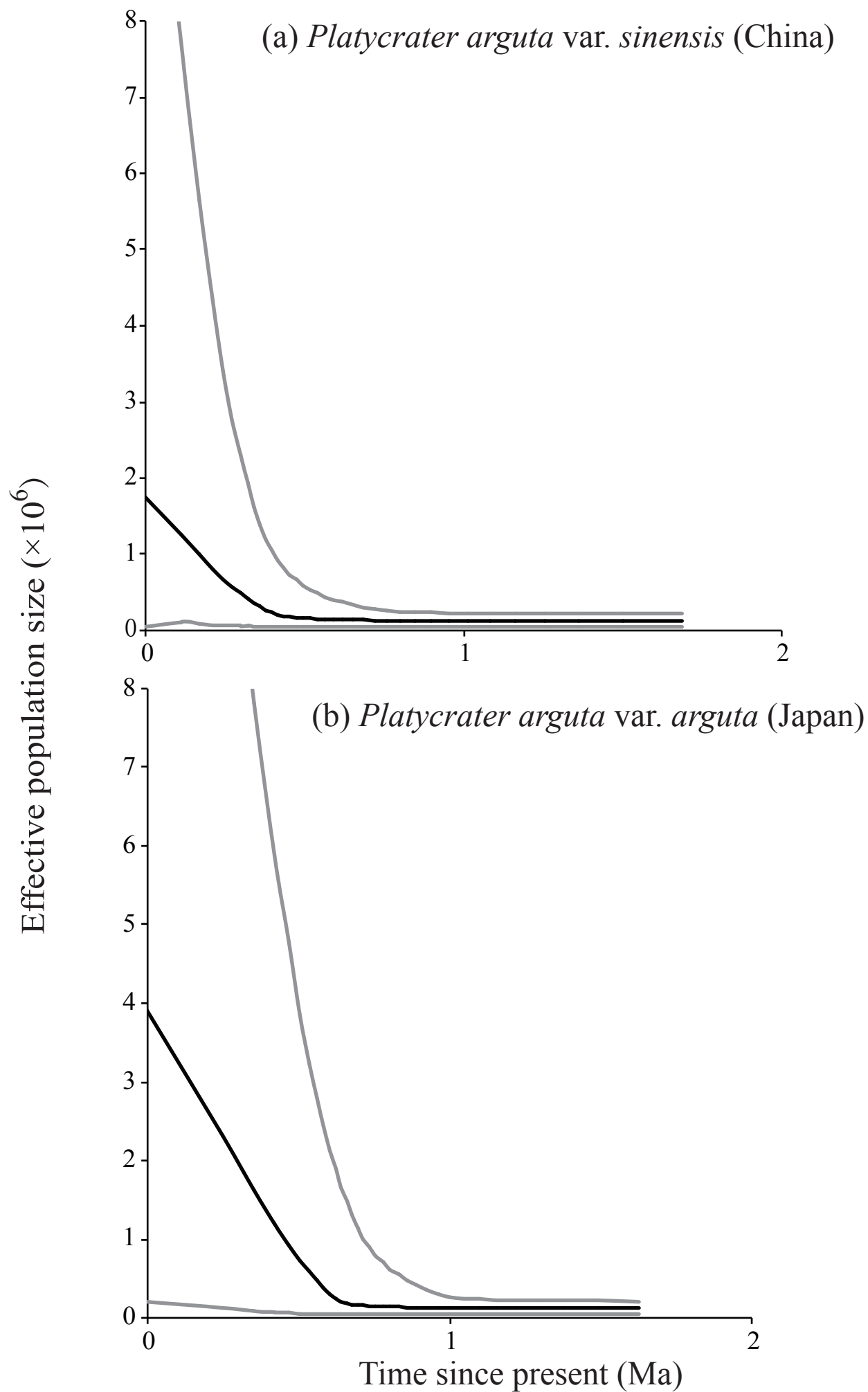

Supplement: Additional file 10: Figure S3 — Extended Bayesian Skyline Plots (EBSPs), inferred from cpDNA and nDNA (ITS, Tpi) sequence variation and depicting changes in effective population size (Ne) as a function of time for (a) Platycrater arguta var. sinensis (East China) and (b) var. arguta (South Japan). The thick solid black line is the median estimate, and the area delimited by the upper and lower grey lines represents the HPD 95% confidence intervals for Ne. [file 1471-2148-14-41-S10.pdf]
