# Supplementary material for: A strong ‘filter’ effect of the East China Sea land bridge for East Asia’s temperate plant species: inferences from molecular phylogeography and ecological niche modelling of Platycrater arguta (Hydrangeaceae)
Source: BMC Evol Biol. 2014 Mar 4;14:41. doi: 10.1186/1471-2148-14-41 (PMC4015774; doi:10.1186/1471-2148-14-41)
Supplement: Additional file 8: Table S7 — BOTTLENECK analyses of nSSRs for 14 populations of Platycrater arguta from East China (var. sinensis: C1–C7) and South Japan (var. arguta; J1–J7). P values are shown for Wilcoxon’s signed rank test, under both the stepwise mutation model (SMM) and the two-phase mutation model (TPM), along with the distribution shape of alleles inferred from the mode-shift test. Population codes are identified in Table S1. [file 1471-2148-14-41-S8.docx]

**Additional file 8: Table S7.** Bottleneck analyses of nSSRs for 14 populations of *Platycrater arguta* from East China (var. *sinensis*: C1–C7) and South Japan (var. *arguta*; J1–J7). *P* values are shown for Wilcoxon’s signed rank test, under both the stepwise mutation model (SMM) and the two-phase mutation model (TPM), along with the distribution shape of alleles inferred from the mode-shift test. Population codes are identified in Table S1.

| Population code |  | Wilcoxon’s sign-rank test | | |  | Mode-shift test |
| --- | --- | --- | --- | --- | --- | --- |
|  |  | SMM |  | TPM |  |  |
| C1 |  | 0.852 |  | 0.711 |  | L-shape mode |
| C2 |  | 0.988 |  | 0.961 |  | L-shape mode |
| C3 |  | 0.980 |  | 0.973 |  | L-shape mode |
| C4 |  | 0.711 |  | 0.656 |  | L-shape mode |
| C5 |  | 0.988 |  | 0.988 |  | L-shape mode |
| C6 |  | 0.988 |  | 0.988 |  | L-shape mode |
| C7 |  | 0.996 |  | 0.992 |  | L-shape mode |
| J1 |  | 1.000 |  | 1.000 |  | L-shape mode |
| J2 |  | 0.766 |  | 0.813 |  | **shifted mode** |
| J3 |  | 0.961 |  | 0.961 |  | L-shape mode |
| J4 |  | **0.039** |  | **0.020** |  | L-shape mode |
| J5 |  | 0.852 |  | 0.852 |  | L-shape mode |
| J6 |  | **0.016** |  | **0.016** |  | **shifted mode** |
| J7 |  | 0.973 |  | 0.973 |  | L-shape mode |

Significant results for Wilcoxon’s test are in bold. L-shaped distribution of alleles is expected in the absence of a bottleneck, whereas a distribution with a shifted mode is expected in a population that has gone through a bottleneck.
